# A Review on the Trends in Event Detection by Analyzing Social Media Platforms’ Data

**DOI:** 10.3390/s22124531

**Published:** 2022-06-15

**Authors:** Motahara Sabah Mredula, Noyon Dey, Md. Sazzadur Rahman, Imtiaz Mahmud, You-Ze Cho

**Affiliations:** 1 Institute of Information Technology, Jahangirnagar University, Savar 1342, Bangladesh; mmredula12@gmail.com (M.S.M.); noyondey8@gmail.com (N.D.); 2 School of Electronic and Electrical Engineering, Kyungpook National University, Daegu 41566, Korea; imtiaz@knu.ac.kr

**Keywords:** deep machine learning, event detection, review, social sensing, shallow machine learning, social media

## Abstract

Social media platforms have many users who share their thoughts and use these platforms to organize various events collectively. However, different upsetting incidents have occurred in recent years by taking advantage of social media, raising significant concerns. Therefore, considerable research has been carried out to detect any disturbing event and take appropriate measures. This review paper presents a thorough survey to acquire in-depth knowledge about the current research in this field and provide a guideline for future research. We systematically review 67 articles on event detection by sensing social media data from the last decade. We summarize their event detection techniques, tools, technologies, datasets, performance metrics, etc. The reviewed papers mainly address the detection of events, such as natural disasters, traffic, sports, real-time events, and some others. As these detected events can quickly provide an overview of the overall condition of the society, they can significantly help in scrutinizing events disrupting social security. We found that compatibility with different languages, spelling, and dialects is one of the vital challenges the event detection algorithms face. On the other hand, the event detection algorithms need to be robust to process different media, such as texts, images, videos, and locations. We outline that the event detection techniques compatible with heterogeneous data, language, and the platform are still missing. Moreover, the event and its location with a 24 × 7 real-time detection system will bolster the overall event detection performance.

## 1. Introduction

Event detection from social media data captures various events happening in real time or events that will occur. This event detection poses three critical questions: what (i.e., name of the event), where (i.e., location of the event), and when (i.e., time of the event). Many people are using social media sites worldwide, and discussions on these platforms also range across various topics. Hence, social media has become the largest ever virtual sensor as users’ data, opinions, location, and so on can be sensed virtually. According to recent statistics for 2021, approximately 4.48 billion people around the globe use social media, which means social media users form almost 57% of the world’s population [1]. With these vast online participants, it is relatively easier to reach that large number of people using social media since they are interconnected. This massive number of people express different opinions about different topics on social media platforms. People post about an event before its occurrence or during that event. Events in social media range across political, cultural, religious, sporting, natural disasters, traffic events, et cetera. Moreover, not all the events pose security threats, for example, cultural events. Therefore, it is vital to gather the maximum possible information about an event to categorize it and take the necessary steps in response properly.

**Figure 1:**
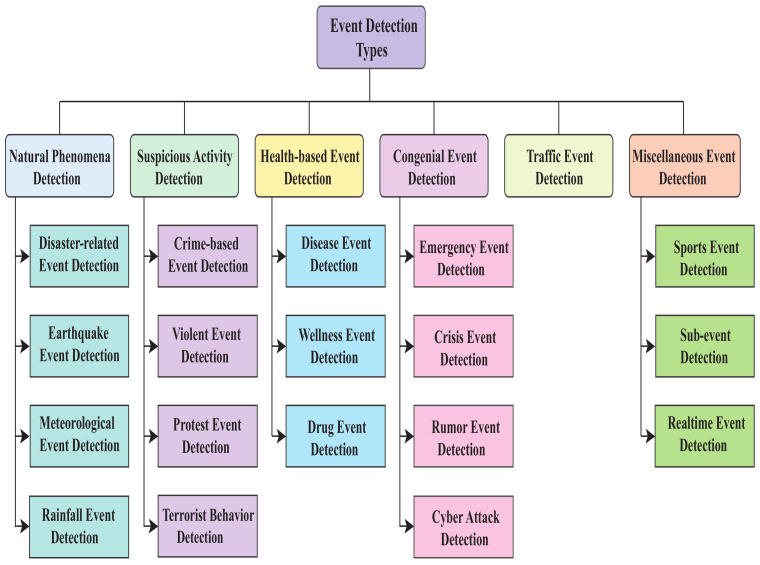
Different varieties of detected events observed in our studied articles during the survey of event detection from social media data.

Many research works have been carried out regarding event detection or prediction from social media over the years because of the rise in disruptive events organized through social media worldwide. In 2018, road safety protests occurred in Bangladesh [2], leading to many disruptive events, especially in the capital of Bangladesh. Dey et al. [3] monitored those events closely on Facebook and proposed a method for event prediction from Bengali Facebook posts. Similarly, researchers around the world have detected events such as diseases [4,5], sports [6], rumors [7], disasters [8,9,10], and so on by analyzing social media content. Figure 1 shows the various kinds of detected events from our studied articles. Interestingly, these event detection studies were performed worldwide, considering different languages. It also indicates how important this study has become for maintaining social security.

**Figure 2:**
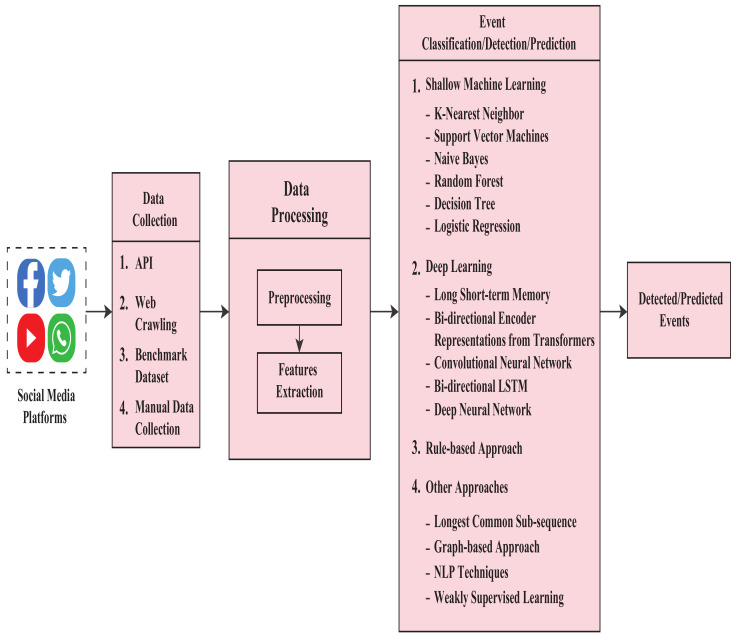
A generic approach illustrating the phases to identify events by employing social media data.

Maintaining security from the analysis of social media data has been challenging since social media contains different data types such as text, images, and videos. Researchers have employed various methods in analyzing these data. Although there are some differences in the methodologies, they all follow the general model shown in Figure 2. Data collection, preprocessing, processing, classification, and visualization are the general steps in this event detection method. In this field of study, most works are based on the collected textual data. That is why it is far more challenging to extract meaningful information from those texts, and hence a data preprocessing step is widely followed. The preprocessed data then move on to the processing steps. The processing steps include different algorithms and other techniques for extracting features used to detect or predict events. Depending on these features, contents are classified as events or not. The last step is to visualize the result, and this visualization can be numeric, textual, or graph-based.

We have encountered some review papers regarding event detection in social media during our study. Some of these reviews only provide a brief description of the previous event detection techniques [11,12,13,14]. Event detection papers include machine learning, rule-based, and other methods. Very few researchers have categorized existing event detection techniques according to their nature in their review works [15]. Researchers have used different datasets for conducting event detection tasks per their needs. A couple of scientists give us a synopsis of these datasets utilized in this domain [13,15]. Event detection from social media data has various genuine applications, including different security purposes. A few researchers have also presented these application domains [16,17]. However, no researchers have provided a detailed analysis of the existing event detection techniques, to the best of our knowledge. No research has focused on the data collection domain, used languages, and extracted features of existing event detection papers. Moreover, none of them have analyzed the challenges in this realm. Therefore, we are motivated to write this review paper. In this paper, we explore several event detection articles and provide a detailed explanation of the used techniques and the main objectives of these articles.

This study reviews different techniques applied to detect events from social media data. It systematically examines the strategies and performance, data collection methods, area, and languages and systematically validates their performances. The main contributions of this paper are described below:

- This review paper considers different techniques ranging across machine learning, deep learning, natural language processing (NLP), latent Dirichlet allocation (LDA), and others in detecting events from social media data. This is the first study to have incorporated such massive techniques in this field.
- A total of 67 research articles from the last decade from different renowned databases were studied.
- Detailed descriptions of the methodologies are provided.
- This study provides information about the datasets used by the respective authors. It also shows their duration of data collection, relative size, data collection area, and, most importantly, what different methods they used for their data collection.
- Different methods’ performance comparison is also provided in this study, which helps understand the actual performance among the research works.
- We identify current research gaps in this field, and at the end of this paper, we also provide directions for future works in overcoming the mentioned gaps.

The rest of the paper is structured as follows: Our research procedure is described in Section 2, and different approaches to event detection are discussed in Section 3. Section 4 discusses the performances of our studied articles. Discussion on the results and future scopes of event detection is mentioned in Section 5, and, finally, this research work is concluded in Section 6.

## 2. Research Procedure

This article reviewed recent event detection work that used social media data. First, we gathered 120 papers from various sources. These sources included Google Scholar, IEEE Explorer, Science Direct, and other scholarly article search engines. Totals of 70, 20, and 30 of the publications we obtained were from Google Scholar, IEEE Explorer, and Science Direct, respectively. We went over the abstracts of these publications after collecting them, and deleted irrelevant ones. Duplicate papers were also eliminated. As a result, we had 67 distinct publications on social media event detection. We thoroughly examined these 67 publications and identified research gaps in the event detection domain. This review primarily focuses on elaborately showing these gaps for future research works. We divided the papers into four categories to provide a full overview of these publications’ working procedures. The articles were categorized as shallow-machine-learning-based, deep-machine-learning-based, rule-based, and other approaches. This classification was made based on the methods utilized. Support vector machine (SVM), K-nearest neighbor (KNN), naïve Bayes (NB), random forest (RF), etc., were classified as shallow-ML-based approaches. In contrast, long short-term memory (LSTM), convolutional neural network (CNN), deep neural network (DNN), and recurrent neural network (RNN) were classified as deep-ML-based approaches. Rule-based approaches include rule-based methods along with rule-based classifiers. Clustering algorithms, the term interestingness technique, and the graph-based process were all included in the other approaches section. These studies covered a wide range of event detection topics: disease events, natural disaster events, rumor events, journalistic events, wellness events, traffic events, city events, etc. Following the categorization, we merged similar types of event detection articles in the same paragraph and linked them. After that, we evaluated the performance of these articles. Tables and bar charts are used to depict the performance evaluations. We also include a qualitative assessment using the Delphi technique, which ultimately provides an overall comparison of our studied articles.

**Figure 3:**
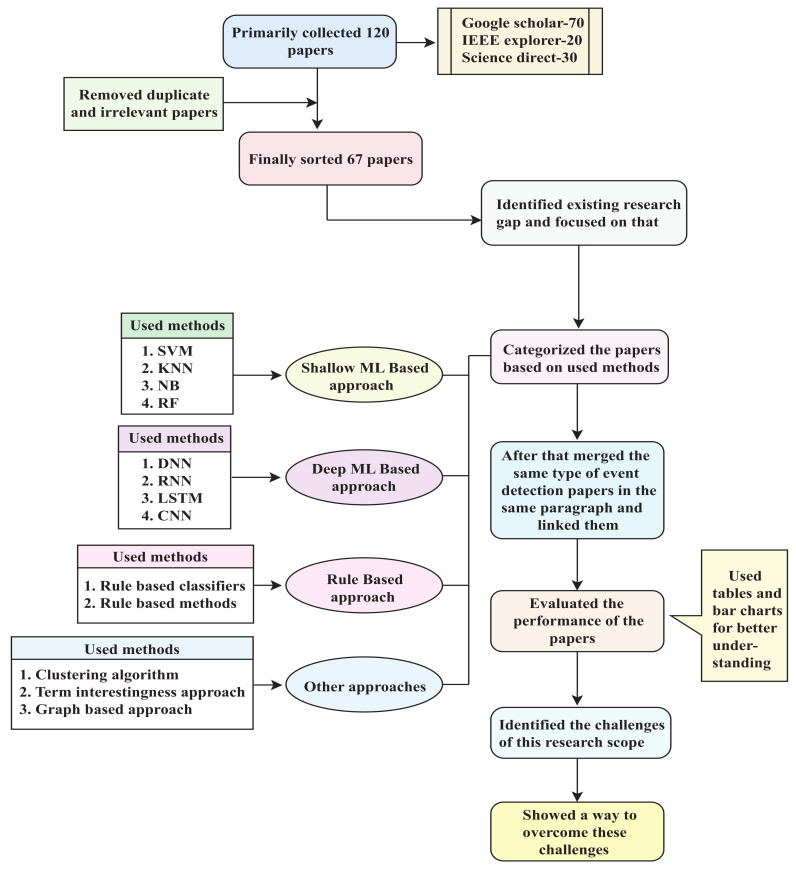
A flowchart visualizing every stage of our research survey from the beginning until the end.

Finally, we highlight the remaining challenges in this study domain and demonstrate how to address them. Figure 3 depicts the flowchart of our research procedure.

## 3. Approaches for Event Detection

In this section, a detailed description of all the articles is presented. This detailed discussion includes their associated models, their analyzed data, selected features, etc. Different approaches such as shallow machine learning (ML), deep ML, rule-based, and so on were adopted in this event detection task. Shallow ML includes methods such as SVM, KNN, RF, NB, etc. Deep ML algorithms include DNN, RNN, LSTM, CNN, etc. In the other approaches, sensor tree propagation, microblog clique (MC), bursty event detection in Twitter streams (BEaTS), and so on were used. The following subsections describe the articles adopting these approaches in detecting events.

### 3.1. Shallow-ML-Based Approaches

Shallow-ML-based approaches are mainly supervised learning methods. An event’s characterization is predefined in these methods, and events are detected or predicted using the predefined labels or characteristics. Hence, these approaches are suitable for specific domains such as celebrating, protesting, religious, disaster, etc. On the other hand, these shallow ML methods are not ideal for new fields except those defined earlier. They cannot learn from the new events. For this reason, these approaches are not appropriate for unsupervised clustering, or they can not work without predefined attributes. Conventional machine learning algorithms such as SVM, random forest, KNN, NB, etc., are examples of shallow machine learning. The event detection articles utilizing shallow ML approaches are described in this portion. In addition, Table 1 describes features, languages, and evaluation metrics used in the shallow ML approaches.

The most promising and vital sector of event detection is detecting disruptive events. It is essential to maintain the overall social balance and harmony. Researchers have been using social media to detect disruptive events. Alsaedi and Burnap [18] proposed a novel disruptive event detection approach from real-time Twitter data. The model took Twitter posts as input, and after processing, it created a table of the detected events of a specific region with sub-events at a particular time. The model preprocessed posts using stemming and stopword elimination processes, then the post was applied to the NB classifier for separating event and non-event related tweets. Next, tweet features were calculated and applied to an online clustering algorithm for generating clusters. For this clustering, the tweet’s similarity was calculated with the already-existing clusters and then assigned a group based on the calculation. Dey et al. [3] also proposed a model for predicting events (celebrating, protesting, religious, and neutral) from Bengali Facebook posts using Bernoulli naïve Bayes (BNB) classifier. Hence, the collected Bengali Facebook posts were cleaned using preprocessing steps (tokenization and stopwords removal). Preprocessed data were applied to the features extraction process, and they extracted common and event-specific word and phrase frequency along with sentiment score. Eventually, all these features were applied to the BNB model for predicting event types. Similarly, Hossny and Mitchell [19] proposed a language-independent method that utilized commonly used Twitter keywords for events such as protests, using a binary classification model. They used keywords from word pairs and a spike detection filter to identify context from the preprocessed data. They binarized the event count vector and every word-pair vector. Finally, they used the Jaccard similarity metric for measuring similarity. The highest valued word pairs were used as features for model training, and eventually, they trained the NB classifier with the extracted features. For detecting local violent events in South Africa, Kotzé et al. [20] proposed an approach by analyzing Whatsapp messages employing machine learning, and showed how local violent events could be recorded and coded over time. They applied LR with unigram and Word2Vec features in detecting those events. At first, they preprocessed the data by removing stopwords, cleaning, tokenization, and syntactic parsing, and then used the NLTK stopwords list for the English language and translated English stopwords into their appropriate African words. For annotation purposes, they used four annotations: land grab, farm attack, protest, and crime.

Ristea et al. [21] proposed a model for predicting crime-related occurrences on sporting days using Twitter data. They applied feature selection models for selecting suitable variables. Then, they applied a localized kernel density estimation model to predict seven crime events. For crime events detection, they extracted hate- or violent-related tweets; hence, they used terms from Hatebase [22]. They analyzed the sentiment of those tweets using the NRC lexicon [23,24]. They applied random forest (RF) classification for feature selection and eliminated features with an importance coefficient value less than 0.05. For crime prediction, they created fixed-size grids, pointed to different points as non-occurrence of crime (NONE), and collected points T from the collected crime event’s location. If T and NONE coincide, then the NONE label is removed. This task was performed by applying a binary classifier. To find additional features of the T point, they applied kernel density estimation (KDE) for spatial features and localized kernel density estimation (LKDE) for historical crime density features.

Traffic events are as crucial as disruptive events since they often turn into disruptive events. Many researchers have used this detection system to detect congestion around a city, traffic conditions around a specific location, etc. Suma et al. [25] detected spatiotemporal traffic-related events along with location and time in the city of London from Twitter data and used big data and machine learning approaches. For this analysis, they collected Twitter data along with the posting time, user coordinates, and postal code. After collection, they cleaned the data by removing URLs, stopwords, mentions, etc., for efficient processing. After preprocessing, they used logistic regression (LR) with stochastic gradient descent for classifying traffic- and non-traffic-related tweets. They also visualized their outcome using Tableau [26] with specific locations. Furthermore, Suma et al. [27] proposed a model for detecting spatiotemporal congestion-related events from London city’s traffic-related Twitter data using big data, high-performance computing (HPC), and AI platforms (i.e., Tableau and Spark [26,28]).

They detected two events: targeted (i.e., traffic) and general, where supervised ML was used for a targeted event, and word frequency analysis was used for general event detection. For targeted event detection, they used LR. They automatically detected many events, such as Underbelly Festival [29], The Luna Cinema [30], etc., along with those events’ locations and times. They integrated big data HPC and implemented an automatic validation. For locating tweeters, they employed Google Maps Geocoding API [31]. It converted latitude and longitude information into human-readable form. Similarly, Alomari et al. [32] proposed Iktishaf, a system for detecting traffic events from Twitter streams in Saudi Arabia. Their system presented hybrid human–ML methods that combined AI, big data, and human cognition. They used three ML algorithms to build multiple classifiers and detected eight types of events to detect events. Collected data were preprocessed and divided into labeled and unlabeled groups. Then features were extracted from the text using the term frequency–inverse document frequency (TF-IDF) method. Eventually, their systems detected events, including a fire accident in Riyadh, rains in Taif and Makkah, the KSA national day, etc., without any prior information about those events.

Natural-disaster-related event detection is another type of disruptive event, and it has been one of the most concerning from the beginning of social media. Hence, a considerable amount of research is being conducted to detect these events using social media data. Sakaki et al. [33] proposed a model for earthquake detection along with center and trajectory by processing Twitter data. They implemented an SVM classifier using keywords, context, and the number of words as features. Twitter users were used as sensors and applied particle [34,35] and Kalman filtering along with Global Positioning System (GPS) data for location information extraction. Later, they classified tweets as event-related or not, and after that classification task, they applied their probabilistic model for event and location detection. Their event detection probability threshold was 0.95. On the other hand, Pekar et al. [36] proposed an approach that can detect real-world disaster events from social media data without any prior knowledge about the events. They tackled this issue by implementing a new ensemble method that utilized the available data on the training dataset. They worked on four classification problems, namely, relatedness, informativeness, eyewitnesses, and topics of messages. This detection process collected data and preprocessed them by removing unnecessary parts, i.e., hashtags, mentions, etc., then used parts of speech tagging. Eventually, they classified the tweets using the ML classifiers. In addition, Spruce et al. [37] proposed a model for detecting impactful rainfall events using social sensing and compared their result with a dataset manually created by Met Office [38]. They studied both English and non-English tweets from various geo258 graphical regions. Met Office events were given severity values in the range 1 to 4, where 4 is the most severe event and 1 is the least severe event. They applied retweets and quotes, bot filtering, weather station filtering, phrase filtering, and ML filtering to keep relevant tweets.

In recent times, event detection is also contributing to the health sector. Social media data are being frequently used to detect various diseases. Cui et al. [4] proposed a model for detecting foodborne diseases and their locations from Weibo data. Hence, they extracted key phrases and used an SVM classifier. They selected ten features by analyzing government reports and using the word2vec library. Then, they calculated the semantic similarities of tweets in vector space. Finally, they used the TextRank algorithm [39] for extracting keyphrases from the tweet, and extracted features were then used for SVM model training. For location estimation, they used various approaches. If the restaurant name was written in the tweet, they used the DianPing website (https://www.dianping.com/ (accessed on 10 April 2022)), which provides its location. If the food name was written in a tweet, they found all the restaurants that make the food using Baidu API. Additionally, they also collected geolocation and the user’s registered location for further information. After detecting events and locations, they also found the other restaurants with the same possibility and scored them. They used this scoring for the recommendation system. Similarly, Joshi et al. [5] proposed a four-step architecture for early detection of acute disease events from Twitter. Data collection was the first step, and they collected data using keywords. In the second step, they removed duplicates, and in the third step, they performed health mention classification utilizing three classifiers. They used two approaches for personal health mention classification, i.e., a heuristic-based classification, rule-based, and a statistical approach. In the statistical approach, they used word embeddings using GloVe [40]. They trained two classifiers using the word embeddings as features, namely, LIBSVM and SVM Perf [41,42]. The final step was the monitoring algorithm, which computed the adapted expected weighted average of time between event values. They observed that their system could detect events before the official time in three cases, and in five cases, their system detected before the first news reporting time.

Apart from the health sector, the media sector is not out of the scope of event detection. Different journalistic events are being identified by analyzing social media platform data. For example, Guimarães et al. [43] proposed a model which could automatically detect journalistically relevant events from social media data. They proposed two approaches, namely, automatic and human approaches. In the human annotation approach, using Crowdflower [44], they detected 9000 posts using a set of filters. For human approach detection, they built CrowdFlower HIT (Human Intelligent Task). They also measured user agreement rate deviation, user’s correct words percentage, and user’s consistency regarding news relevance. The automatic annotation approach considered news content and social media posts’ similarities to detect their relevance. On the other hand, Kolya et al. [45] proposed a monolingual event or topic tracking system for the Bengali language using newspaper corpus from the web archive. Their intention was to find out whether two news documents of the same period reported the same event. Hence, they described an event as a collection of vectors consisting of term, location, person, time, date, and organization. Event vectors were generated using the SVM-based named entity recognition (NER) system. They considered a threshold vector value to find out whether the total number of vectors of two separate documents matched by at least this threshold. If the two documents matched or crossed that threshold value, events were detected as the same event. This tracking system selected any news documents of a particular date as an initial story, and news documents of the preceding 15 and following 15 days were considered the target stories.

Rumors are also frequently seen in the media sector, and they were also detected using social media data. Kumar and Sangwan [7] presented a basic elementary concept of rumor detection, i.e., what, why, and how, in social media. Different types of rumors and the process of detecting rumors in social media were also present in their work. According to them, information could be of two types: a non-rumor and a rumor; then, the rumor could be classified as unspecified, true, or false. A false rumor was further classified as misinformation and disinformation, including humor, hoax, finger-pointing, tabloids, and yellow press. Using different machine learning approaches such as NB and SVM, they classified their dataset as rumor or non-rumor.

Besides detecting main events, sub-events were also detected by the researchers. Nolasco and Oliveira [46] presented a framework that could detect sub-events from prime events in social media. They used LDA in their work of sub-event detection. After the identification of sub-events, they performed label generation. For this, they first selected a list of candidates, then scored these labels using a matrix, and finally, they made the label selection.

Shallow ML techniques have also been used in real-time systems. Feng et al. [47] presented a real-time approach for detecting events from social media streams. They used locality-sensitive hashing (LSH) to implement their work. With the help of part-of-speech (POS) tagging and the SVM classifier, they also presented a location extraction process that can extract message-mentioned locations. The first computed the similarity of the newly arriving message with every message presented in its candidate set using LSH. If the matching scores of this comparison were greater than a predefined threshold value, then the new message was placed into the same cluster where its nearest neighbor resides.

Researchers detected not only global events but also local events. Zhang et al. [48] proposed a method for online local event detection and named it “TrioVecEvent”. Their proposed approach was a two-step detection technique that detected events from geotagged tweets contents. Firstly, it divided the tweets in the query window and formed geotopic clusters. In this online clustering approach, they developed a Bayesian mixture model which combined semantic embeddings and geolocations. Secondly, considering the clusters as candidate events, their approach extracted a set of features to classify the candidate events.

Apart from the events mentioned above, different researchers used social media for other event detection. Some determined the trustworthiness of a specific event, some identified semantic meaning of an event, some extracted incident information from events, and so on. Hence, Jain et al. [49] proposed a weakly supervised technique that considered strong and weak event indicators along with implicit event indicators, such as actors, props, precursor events, etc., to detect events from Twitter posts. Their model was capable of learning event indicators automatically, and it associated bootstrapping and clustering of the event indicators as the two main steps. Event-related tweets and their implicit event indicators were identified from the tweet, and the specificity factor was calculated, which determined the event indicator types. On the other hand, they used the affinity propagation algorithm for the clustering process, identifying the context of the specific tweet. Additionally, the frequency of words and the sum of the word embeddings were also used for clustering. On the other hand, Bodnar et al. [50] proposed a model of veracity assessment for information distribution on Facebook and Twitter by using NLP and ML. They determined the trustworthiness of a specific user by processing his or her social network profile and, hence, tried to find out the actuality of a discussed topic by a user. To find the reality, they first transformed the user profile into a feature vector representation, and then they applied a classifier for accurate event detection. They collected both numerical and textual data from the user. The event was considered real if the user’s claim matched the real event occurrence. They tested their data with eleven ML classifiers and selected the one with the best result. Abebe et al. [51] focused on the meaning of the event rather than veracity assessment and, hence, proposed a model named Social-based Event Detection, Description, and Linkage (SEDDaL) for detecting events and identifying the semantic meaning of social media objects. They collected data from heterogeneous social media platforms, i.e., YouTube, Twitter, and Flickr, and generated semantically meaningful interconnected events. Four central modules support the SEDDaL model: (1) Description of social media objects using Metadata Representation Space Model (MRSM); (2) Similarity measurements of objects following MRSM; (3) Event detection using adapted unsupervised learning algorithm; (4) Capturing the metric, directional, and topological relationship between events. MRSM found objects’ temporal, spatial, and semantic features and defined an event with a unique id, temporal, spatial, and semantic coverage representations. On the contrary, a hot event evolvement model, named the Hot Event Evolution (HEE) model, was presented by PN and GEORGE [8]. To avoid data sparsity, they combined short texts of microblogging data into a single document. Their model made clusters of similar types of microblogging comments and yielded a summary so that the user could easily understand the main content of the comment list without reading the whole list. A hot event automatic filtering algorithm and an automatic topic clustering algorithm were used to implement their research work. Apart from the user and their events’ assessments, Gu et al. [52] were interested in extracting incidents from social media. They initially used a dictionary of keywords for collecting data, and, gradually, this dictionary was expanded iteratively. Collected tweets were classified into two categories, either traffic incident (TI) or non-traffic incident (NTI), using a trained semi naïve Bayes (SNB) classifier. All the tweets classified as TI were then sent to a geoparser and geocoder to identify their location. The output TI tweets from the geocoder were again classified using a supervised LDA classifier. This classifier labeled the tweets into five predefined categories: accidents, road work, hazards and weather, events, and obstacle vehicles. Similar event extraction was performed by Nguyen and Jung [53], and for this, they proposed a method for event extraction along with event tracking from social media data. They used the term frequency–inverse document frequency (TF-IDF) method, and while using this method, they considered a group of messages into a single document. They collected data from Twitter using a predefined search condition and then removed unnecessary information and stored it in JSON format. Then, they analyzed these tweets to extract terms from them. The terms occurrences and diffusion information were used for creating signals. After that was the feature extraction and the clustering phase, where they applied density-based clustering on these tweets. Bide and Dhage [54] presented an effective model named Similar Event Evolution Detection (SEDM) for detecting similar events based on the event’s temporal behavior. They also proposed a self-tuning clustering algorithm for combining tweet clusters of similar contents. Event clusters were generated by similarity matrix calculation. Their experiment first clustered the microblogging events together to identify their similarity and keep track of these events. With the help of this information, they further predicted another event topic that lies under the same class.

### 3.2. Deep-Machine-Learning-Based Approaches

Deep-ML-based methods are unsupervised approaches. They learn from the given data; hence, they can be used to categorize different events or topics. For this reason, these approaches are useful to detect multiple events in place of focusing on specific domains. In addition, events’ characteristics do not need to be predefined, hence saving a lot of time and space for the calculations. On the contrary, events or topics can be misidentified or ambiguously classified into a cluster if that event’s instances are small. For this reason, a sufficient number of instances are needed for training in using deep ML approaches. DNN, RNN, LSTM, and CNN are some examples of deep ML. In this portion, we describe event detection articles that utilized deep ML algorithms. Additionally, Table 2 describes the features, languages, and evaluation metrics used in the deep ML approaches.

Deep ML techniques have been widely used in traffic event detection tasks. This is evident where Zhang et al. [55] proposed a deep-learning-based traffic event detection system from Twitter data. They used paired tokens and employed LSTM and deep belief network (DBN) on the extracted tokens. Their model followed three steps. At first, they extracted individual and paired tokens from the text using a systematic feature selection process. Hence, they identified traffic-accident-related tweets using commonly used keywords and preprocessed them by removing stopwords, and then stemmed them using Porter stemmer [56]. In the end, they used appropriate stemmed tokens as features. They also used paired tokens which were selected using the association rules, i.e., Apriori algorithm [57], between stemmed tokens and manual labels. Secondly, they validated the effectiveness of their model. They thirdly presented the advantages and disadvantages of the traffic event detection process by comparing loop detectors and accident logs from the state Department of Transportation and traffic data.

Deep ML techniques have also been used in detecting disaster-related events. Among them, Shi et al. [58] specifically used these techniques for early meteorological event detection and proposed a model named Sentence-level Feature-based Meteorological Event Detection (SFMED). Their model was composed of two modules: the first module was a fine-tuned bidirectional encoder representations from transformers (BERT, i.e., BERT-base, Chinese) for language model and the second module was for event detection, named a wide-grained capsule network. Every Weibo post was transformed into a sentence vector by the BERT model. Corresponding features were learned by the capsule network (CN) and the post was classified into meteorological events. Burel and Alani [59] proposed a web API named Crisis Event Extraction Service (CRESS), and it could automatically classify crisis-related documents from social media. It could extract event-related documents, types, i.e., floods, haze, etc., and information categories, i.e., affected individuals, donations, volunteers, etc. For these three types of information extraction, they built three CNN-based models. At first, they cleaned the collected data and created pretrained word embeddings matrices from the tokens using the Word2Vec model. Those matrices were then used for training the CNN model. Abavisani et al. [60] also proposed a novel crisis event detection approach that considered both image and text for classifying crisis-related events. For any given text and image pair data, they first generated a feature map from the image and created word embeddings from the text. They fused extracted information using a cross-attention mechanism. They introduced a multimodal variety of stochastic shared embeddings (SSE) to regularize the training phase and deal with limited training data. In short, their approach found out whether the data from social media were related to crisis events, and if so, then which type of event it was, and finally measured the severity of the detected events. Additionally, Imran et al. [9] presented a framework that could be used for the extraction of disaster-related valuable information from social media. Their proposed system consists of the two-steps classification of tweets and extraction from those tweets. For classification purposes, they separated messages into two main classes—personal and informative. They further classified informative messages into two types, named direct and indirect. After the classification of a tweet, class-relevant information was extracted. They used a machine-learning algorithm named conditional random field (CRF) for their work.

Additionally, Fan et al. [61] proposed a hybrid machine learning approach that disclosed disaster events of different locations from social media data. Their proposed pipeline consisted of three basic modules: input, learning, and output. The input segment mainly prepared the social media content for learning. The learning module was composed of two elements. The first one used NER and Google Map Geocoding API to identify the posts’ location entities, and the second one used BERT for classifying the posts into different categories. The output module was used to interpret and anticipate the learning module results.

Furthermore, Huang et al. [62] proposed a new approach for detecting emergency events such as public health events, natural disasters, social security events, and accidents from social media data. Their proposed method mainly clustered the social media contents based on 3W, i.e., what, when, and where. They considered four tasks for detecting emergency events. These tasks were emergency- vs. non-emergency-related posts, types of these emergency events, attribute information of these events, and clustering of the posts. Using the BiLSTM method, they extracted the time- and location-related information. Finally, text similarity was calculated based on event type, location, and time in the clustering phase.

Researchers also emphasized sports events, along with disaster and traffic events. Such is seen where Kannan et al. [6] presented a structure for detecting events from the sports domain. They used LSH techniques in their work. They also introduced a completely new event lexicon of cricket sports that could represent 37 key events of this domain. This lexicon was enriched with various terminologies, which were collected from ESPNCricInfo. A cluster was deleted after the detection of an event from it. For detecting an event from a cluster, they checked if the posted rate of the target cluster exceeded the predefined threshold value or not. To prevent duplicate event alerts, they compared the timestamps of the new event cluster with the timestamps of the already existing cluster. The new cluster was discarded if this difference was less than 60 s.

Health issues are given paramount importance nowadays, and social media data have also been used to detect health-related events. Shen et al. [63] proposed an adverse drug event (ADE) detection structure from Twitter data. They presented a graph adversary representation (GAR) framework integrating two regularization models to develop ADE detection performance. The two integrated models were data augmentation based on graph embedding and adversarial training. In the pretraining phase of the data augmentation model, the initial text was formulated as a graph structure so that a vast amount of learning samples could be added to the embedding module by sampling through the graph. Adversarial training, another regularization strategy, was utilized to hold the model back from depending on those features which were not biased by summing up adversarial perturbations to the training sample.

Health-related events, disasters, traffic, and other events happen around different geographic regions since social media has vast regional coverage. Hence, it is normal to have different lingual presentations of the same topic in different regions. For this reason, researchers have expanded their work in multiple languages. Their proposed models are suitable for more than a single language. For instance, Liu et al. [64] proposed a multilingual event detection approach, named the multilingual event mining (MLEM) model, that detected events automatically and produced evolution graphs. Hence, they first transformed different languages’ text into a common form, accumulated similar phrases and entities, and applied different similarity measures using the word2vec model. They used the TextRank model by word2vec for generating event summaries. For generating evolution graphs incrementally, they used the line clustering method. Finally, they built an application named RING using this MLEM model. Likewise, Ahmad et al. [65] also proposed an excellent framework for multilingual event recognition in the disaster domain. Their model was based on deep neural networks. Their first model was based on bidirectional long short-term memory (Bi-LSTM) and CNN. With the help of monolingual word embeddings, they trained this model independently for each of the three languages. An additional multilayer perceptron (MLP) technique was used in the final layer of their second model, which was also applied separately for each language.

Events also generate other sub-events, which sometimes greatly impact the actual events. Hence, some researchers also shifted their focus to detecting sub-events from social media data. Bekoulis et al. [66] predicted the existence of a sub-event along with the type of the sub-event from Twitter data. Unlike others, they mainly emphasized the chronological relation of the consecutive tweets. They first divided the data stream into several time slots and then formed bins to hold the tweets. Finally, they evaluated the scenario of each tweet-bins and predicted the existence and the type of each sub-event. For evaluation, two strategies were adopted by them. The relaxed evaluation was where a sub-event was assumed correct if a minimum of one of its bins had been detected as a sub-event. Another one was a bin-level evaluation that is stricter than the previous one, where each bin was counted separately to check whether the prediction was correct.

Apart from the event mentioned above, deep ML techniques have been used to detect other events on social media. Some have detected temporal events while others have tracked events and so on. Chen et al. [67] proposed an online event tracking and detection model using neural networks and similarity metrics. When a tweet arrived, its similarity metric was calculated with the existing tweets. If it already existed, information in the memory was updated, and it created a new id if the event was not in the memory. For similarity metric learning, they employed a function f (T1, T2). It returned a value closer to 1 if the events were similar or a value close to 0 if they were dissimilar. A tweet’s low dimension representation was calculated for event tracking, and an entry from memory was selected using the nearest neighbor. If the computed similarity exceeded the threshold for the selected entry, the tweet was relevant and updated. Otherwise, a new entry was created in the memory. Aldhaheri and Lee [68] proposed a framework for temporal event detection on social media. They implemented their system into Twitter data which was classified into two types, i.e., tweets and retweets, and converted them into temporal images. They first transformed these social media graphs into adjacency matrices and then generated grayscale images from these matrices by using the downsampling technique. They also applied a multilayer neural network to analyze the model’s performance. Qiu et al. [69] proposed a simple but efficient single-pass clustering method for event detection on social media. Firstly, they developed a matching dataset based on the similarity of the tweet–event pair. Then, they grouped the same event-related tweets using the incremental clustering approach. Their model selected several active events from the existing event library whenever a new tweet was encountered. The corresponding similarity of the new tweet and the candidate events was calculated to determine which event group the new tweet should go to. After that, the existing event library was updated. Ali et al. [70] presented a sentence-level multiclass event detection model for the Urdu language. They used deep learning models and word embedding, one-hot encoding, TF, TF/IDF, and dynamic embedding-based feature vectors to evaluate their model’s performance. Their model detected 12 different events. They found the best accuracy with the TF/IDF-based scheme. They also compared their approach with traditional machine learning models, and their DNN approach outperformed others. The preprocessing step removed all the non-Urdu words and sentences, URLs, and special symbols. Additionally, stopwords elimination, tokenization, and sentence filtering were also applied. Generated features were fed into the embedding layer, and output from the embedding layer was fed into the neural network’s fully connected/dense layer.

### 3.3. Rule-Based Approaches

Human-generated rules store and manipulate data in rule-based systems to imitate human intelligence. Hence, a set of rules must be issued for a specific task, and this provides a useful way for a specific domain event detection. Specific domains can be detected easily and in a detailed way, resulting in a high-accuracy system. These rules also make rule-based systems vulnerable to multiple event detections or clustering approaches since rules are not generated dynamically. Hence, a wide range of event detection is not possible with no prior data about them. In this portion, we describe rule-based approach papers.

Rule-based approaches are popular in disruptive event detection. Alhalabi et al. [71] used such an approach, and they proposed a system for detecting terrorist behavior. They also presented an algorithm for anti-terrorism operations. After collecting tweets, they analyzed these tweets’ textual contents and image contents into separate streams. They combined these two contents in such cases where the tweet contained both, i.e., text and image content. In the text stream, the tagging of the input tweets was performed using Ontotext [72]. After that, they applied two decision rules for rating the degree of the suspicious tweet. In the image stream, they first identified the image’s path, then downloaded the image, and finally, they sent the image for further analysis. Thus, combining the stream results, they calculated the probability of suspicious acts.

Real-time systems have been one of the most interesting settings in event detection schemes. Different methods have been used for making real-time systems, and a rule-based approach is one of them. Li et al. [73] proposed a real-time novel event detection method that found out who, what, where, and when terms. Hence, they split the term space of tweets into a collection of terms that had the characteristics of the mentioned aspects. These collections were named semantic classes, presenting one or more event characteristics. Mention, location (using OpenCalais), named entity, verb, hashtag, noun (using TweetNLP [74]), and embedded links were included in these classes. For clustering, they used three methods; retweet, link, and semantic class clustering. They calculated class-wise similarity using TF/IDF and cosine similarity and aggregated for clustering events. To exclude old events, they used a temporal identification module. Novelty score was calculated for each event to compare how novel the event was, and the score was in the range 0–1, where a higher value defined a higher novel event. In addition, Girolamo et al. [75] also proposed a rule-based real-time event detection approach that used online clustering by utilizing the evolutionary game theory along with replicator dynamics. They considered Twitter their data source and experimented with the Twitter data features. They followed Kappa architectural model [76] and Apache Cassandra, a Big Data Analytics platform for implementation purposes. Tweets were preprocessed by applying a rule-based approach, and they applied three rules for preprocessing. These preprocessed tweets were the inputs of the online clustering process.

Rule-based approaches are also used in other, different event detection. Saeed et al. [77] proposed a method named Weighted Dynamic Heartbeat Graph (WDHG) for detecting events from Twitter data. Their model transformed the set of texts into a sequence of temporal graphs, i.e., WDHG. The heartbeat score was calculated from the graph, and aggregated centrality and growth factor were considered features. The aggregated centrality represented different topics’ central tendency and their coherence in WDHG. Created graph nodes’ accumulated weights were defined using growth factor, and it represented the topic’s intensity of drift and popularity in the data. Further, a rule-based classifier was used for labeling the candidate graphs. By following this process, a list of detected topics was presented.

### 3.4. Other Approaches

This subsection describes the event detection articles that did not use shallow machine learning, deep machine learning, or rule-based approaches. In addition, Table 3 describes features, languages, and evaluations metrics used in the other approaches.

Apart from the ML- and rule-based approaches, other methods have been used to detect disrupting events. Ansah et al. [78] proposed a language-independent propagation-tree-based model named SensorTree for detecting protest-type events from Twitter data. It captured the propagation of information in the community and detected the information burst by modeling sudden changes in the flow of information. Hence, SensorTree first took tweet streams as input and created a propagation tree. Tree-related information was kept in a data grid, and then the SensorTree used a burst sensor for computing changes in the propagation tree and detected a burst from the tree. After that, the SensorTree implemented a topic model that extracted events. In addition, a dynamic approach to detecting cyber-attacks from social media was proposed by Khandpur et al. [79]. In this approach, the event was triggered in a weakly supervised method using only a small set of seeds. They first performed the task of target domain generation, where each tweet was converted into its dependency-based tree form. After using these trees, they performed their query expansion. Finally, they had completed the event extraction task. Thus, the final tuple of events was generated. They used two base lines for evaluating their performance. Target domain generation using expectation regularization was their baseline 1 [80], and cyber-attack event detection using bursty keywords was baseline 2 [81].

Road traffic events were also detected using other approaches. For instance, Alomari et al. [82] analyzed road-traffic-related tweets in the Saudi dialect and employed the lexicon and sentiments of the tweets to detect traffic events. They tokenized, normalized, and extracted named entities from the data using the SAP HANA system. Their applied sentiment analysis tried to determine the driver’s emotions. They also extracted location information from tweets directly. Eventually, they detected events by matching words and finding sentiments from the tweets. They scored tweet words based on the four sentiment classes and classified a tweet based on the word’s value, including how many times it occurred in the tweet.

Natural-disaster-related events were detected using other approaches. Rossi et al. [83] used social media content along with probability-based weather prediction for detecting current events and extracting valuable information. Their event detection algorithm returned a binary signal as output. It returned true if any event was found and false if not found. When the result was truly a notification incorporating all the data that caused the algorithm to be true, it was sent to the end-users for authentication until the next weather forecast became available. For collecting the informative tweets they used NLP techniques, e.g., fasttext tool [84,85,86]. For language analysis, they used the CELI proprietary resources [87], and for storage, they used PostgreSQL [88].

Similar efforts have been placed on detecting drug events from social media. Rosa et al. [89] proposed a method for supporting pharmacovigilance activities by analyzing social media data, i.e., Twitter and PubMed. They first applied the automatic harvesting of the collected tweets and PubMed’s paper abstracts. Then they applied a fuzzy formal concept analysis that created two contexts and two formal lattices that demonstrated the correlation between a drug and its reported side effect. The system finally compared these two mediums’ correlations and inspected if the official site, i.e., sidesffects.embl.de, also listed the same side effects. This inspection eventually confirmed an event.

Besides traffic events, city events have been explored and detected using other approaches. Such is evident where Anantharam et al. [90] proposed a model that processed Twitter data of city areas to reveal city events by creating a CRF model automatically. For annotation of multiphase entities, they used BIO notation [91]. The model also created an automatic training that employed domain knowledge of instance-level. This instance-level took into account the city locations, event terms, etc. For location information, they trained the sequence model with information from Open Street Maps (OSM) [92] and 511.org entities. They used spatial, temporal, and thematic coherence to characterize city events and applied an aggression algorithm that extracted events from the annotated text. Eventually, they categorized events as scheduled events, active events, etc.

Events’ sub-events were also detected by Arachie et al. [10], who presented a framework based on unsupervised learning for detecting sub-events from Twitter. Their method was divided into three parts—extracting sub-events, ranking sub-events, and clustering them. For extraction of nouns and verbs, they used the spaCy dependency parser. They applied the Gensim phrase model to identify the sub-events that were not recognized by the noun–verb pair. Using an MOAC ontology containing 62 terms, they compared their candidate sub-events with those terms and calculated the maximum cosine similarity to rank the candidate sub-events effectively. After that, they clustered their candidate sub-events using spectral clustering.

Considering the importance of real-time event detection, other approaches were also employed along with ML- and rule-based approaches. Fedoryszak et al. [93] presented a real-time framework for event detection that was able to detect event clusters, which particularly means the clusters of event-relevant entities, in every minute. They also presented an offline simulation model to reduce the noise impact of these clusters. Using a subjective trend detection structure, they emphasized those clustering entities that seemed to be trending. After clustering entities, they also applied cluster linking, where they discarded low-weighted cluster edges by comparing them with a predefined threshold value. In the case of offline evaluation, they used the same dataset but diverse settings. Then they measured their performance with different matrices such as events detected fraction, clustering quality, merged event fraction, and duplicate event fraction.

Some researchers also dedicated their work to detect local events. Such is seen in the works of Choi et al. [94], where they proposed a local event detection model from Twitter by analyzing users’ posts, comments, threads, and geographical information. Their model was composed of four modules: data collection, graph modeling, relevant analysis, and detection of local events. They used a geographical dictionary for detecting local events from non-geotagged postings, embedded geographical information of posts containing geographical information using text mining, and eventually produced a weighted keyword graph utilizing the characteristics of social networks. A clustering algorithm then detected events using that graph’s edge weight.

Rather than focusing on event detection, some have worked to discover events, event tracking, event clustering, etc. Yang et al. [95] proposed an event discovery approach named In-domain and Cross-domain Laplacian Regularization (ICLR). They considered both text and image for event detection. They preprocessed the collected data to obtain unified feature vectors and used specific dictionaries for each domain. They applied inverse function for measuring similarity between data sample and dictionary base, hierarchy in WordNet for title similarity matching, and TF-IDF for textual similarity. They used the Jaccard index for image tag similarity, and for description similarity, they used WordNet. They extracted the fully connected layer of Places205-AlexNet [96] to recognize the scene. On the other hand, Comito et al. [97] proposed an online algorithm that found out which topics people were interested in over a given period, and they intended to detect emergency events or disruptive events from the Twitter stream. Hence, they employed BEaTS method. The model processed the tweets one at a time and then updated the cluster incrementally. A new tweet would join an already created cluster if it met the given threshold; otherwise, it formed a new cluster. A bursty event was detected if a cluster received a vast amount of tweets in a short period. Similarly, Gao et al. [98] detected events and proposed an approach of a new concept named microblog clique (MC). In this process, a microblog formulated a hypergraph, and bipartition was implemented. For hypergraph, they found textual information using the TF/IDF process; for visual information, they extracted the spatial pyramid feature of two images, employed Harversine formula [99] for measuring geographical similarity, calculated social similarity to find follower/followee relation, and found temporal information by looking at posting time. The created MCs and corresponding microblogs were used to construct a bipartite graph. Finally, to detect events, they performed the bipartite graph partition. Shi et al. [100] also proposed a novel model named Event Detection and Multisource Propagation (EDMP) for detecting events and multisource propagation that learned from previously detected events. First propagation, learning process, and consecutive propagation were the three steps used for intelligently propagation of events. They used the topic-popularity-based event propagation (TPEP) method for detecting topic-popularity-based event propagation since popular topics were propagated fast.

Dong et al. [101] further scaled this event detection process and proposed a multiscale event detection scheme from social media data by considering different spatial and temporal scales of events. They measured the pairwise similarity of tweets using the TF-IDF method and then created an undirected and weighted graph where vertices are tweets and edges with weights represent the similarity score. They used the Louvain method [102] for graph-based clustering, where each cluster was expected to have the same topic’s tweets. Then, they studied the data behavior by applying spatiotemporal statistical analysis, and they defined a term-filtering method in this regard. Hasan et al. [103] presented a framework for detecting events named TwitterNews+ from the Twitter data stream. Their system consisted of two main modules: the search and event cluster modules. The search module promoted similar tweets’ retrieval and yielded a binary verdict about the input tweet’s novelty. If the cosine similarity of an input tweet was greater than a predefined threshold value, then it was nominated for an existing event cluster. If not, then a new cluster was created for the input event. Finally, using longest common subsequence (LCS) on word level and with the help of different filters, they identified newsworthy events. Their target was to detect non-bursty events, which refer to minor events. They used a burst detection approach for this detection. Valkanas and Gunopulos [104] mainly concentrated on the complication of event identification from live web data and proposed an effective approach to tackle this issue. They first clustered the users based on their geographical location for detecting events. Then, they independently monitored the emotional state of each cluster, and at the point where the cumulative emotional state changed suddenly, they treated that point as an event. Each tweet was classified into seven emotional states, named anger, fear, surprise, disgust, happiness, sadness, and a none state. Their main system took Twitter stream as input, and then these inputs were fed into two subsystems—emotions classifiers and the location extraction subsystem. When an event was identified, they noted the termination time of the aggregation interval as the events occurrence time, and the tweet ids of the events were passed to the event extraction mechanism where event summarization was performed. Sun et al. [105] presented a model for detecting events by investigating documents of various microblogging sites. They named their model Efficient eVent dEtection (EVE), which consisted of three components. Firstly, a scoring technique based on the hypertext-induced topic search (HITS) algorithm was introduced to filter the entire dataset. Secondly, they used probabilistic latent semantic analysis (PLSA) based on a probabilistic topic model, which was used by them to find out latent events in the data stream. Finally, the expectation-maximization (EM) algorithm was utilized for training the parameters. Sivaraman et al. [106] introduced a unique parameter for detecting events. They focused on how they could track down a distinct social attribute for tweets involving events without examining the main content of the tweets. They detected events by utilizing Twitter synchrony and the average number of common friends (ACF). They used a term interestingness approach [14] where hashtags were considered as terms. They also proposed an algorithm for calculating ACF. They labeled their data by manually surveying 27 individuals and divided their dataset equally into two halves. Then, they calculated mean (m) and standard deviation (std) for both the datasets, and if the ACF was greater than the value of (m + x ∗ std), then it was counted as an event, otherwise a non-event. This hypothesis was tested with different values of x to obtain the best result. Akbari et al. [107] shifted their focus from traditional event detection and stated that the remaining event extraction methods were not suitable for personal wellness event extraction as the social domain was full of noise and also due to the diversity and interrelation of event-related data. Therefore, to address this problem, an approach that made the best use of social content to extract wellness events and highlighted the interrelation between them was presented. Their proposed framework was also beneficial for some user-health-based applications such as user health profiling, personalized lifestyle assistant, etc. For modeling the relations among events, they proposed a graph-based multitask learning model. The main assumption of this model was that different tasks were supposed to be interrelated with various weights and parameters. On the other hand, Zakharchenko et al. [108] showed that journalists of Ukrainian “high-quality media” do not care about the fact that some media topics are introduced only to manipulate the agenda setting. To demonstrate that, they assembled the data about those publications which targeted such types of topics in “high-quality media” of Ukraine. They also presented their exposure analysis and then compared the outcome of the topic with experts’ assessments of “media quality” and “artificiality rate”. They did not track down connections between the number of publications and the “artificiality” of the topic. Their research highlighted a special case of politician’s manipulation named “pseudo-event”. Further, they focused on a particular type of pseudo-event, namely, “fake newsworthy event” or “fake peg”. They finally postulated two hypotheses; the first one stated that newsmakers utilizing fake newsworthy events effectively support their manipulative themes even into the “high-quality media”, and the notability of these topics does not relate to the indicators of “media quality” and “artificiality rate” of the topic, assessed by the specialists. The second one stated that other newsmakers might stand up to the expansion of topics in view of fake newsworthy events by introducing their own newsworthy events. Again, Pomytkina et al. [109] conducted research, and the motivation behind their mental hypothetical and experimental exploration was to test the techniques for cyberbullying research, to concentrate on self-esteem as a potential determinant of the position and role in the demonstration of violence on the Internet. The point of the research was to observationally recognize the signs of cyberbullying and the nature of its effect on student youth: specifically, figuring out the reasons for utilization of social networks by students; distinguishing students’ roles and dominant positions during cyberbullying; distinguishing the characteristics of youngsters’ reactions to cyberbullying on social networks; development of the connection between the roles and positions filled by students during cyberbullying and their confidence. For this research, they used a set of methods that included theoretical, empirical, and individual interviews and questionnaires. They experimented with over 105 students aged from 18 to 22 of the National Aviation University (Kyiv).

**Table 3:** A brief description of the selected features, studied languages, and evaluation matrices used in articles that applied other approaches.

Ref.	Selected Features	Used Languages	Evaluation Metrics
[82]	×	Arabic, Saudi dialect	
[83]	Semantic features such as proximity expression, lemmatization, exclusion	×	Precision, recall, F1
[89]	×	English	Correlation
[90]	Annotated tweets with event terms and location (for CRF model creating)	English	Precision
[93]	×	English	Events detected fraction, clustering quality, merged event fraction, duplicate event fraction
[97]	×	English	Recall and precision
[106]	×	English tweet	Precision, recall, F1-score
[107]	NGrams, named entities, Gazetteer, and modality	×	Precision, recall, F1-score

## 4. Results

**Figure 4:**
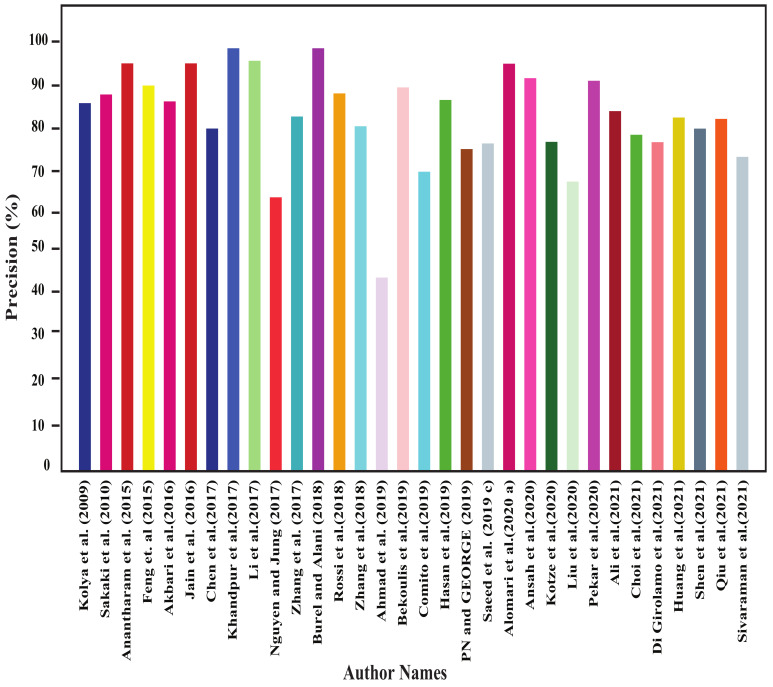
Precision-based performance comparisons of the articles. The height of each bar presents an article’s precision, which is given in the Y-axis, and the X-axis shows the author’s name. Refs. [8,20,32,33,36,45,47,48,49,53,55,59,62,63,64,65,66,67,69,70,73,75,77,78,79,83,90,94,97,103,106,107] have used precision for their performance analysis, and their values are presented using the bars in this figure.

**Figure 5:**
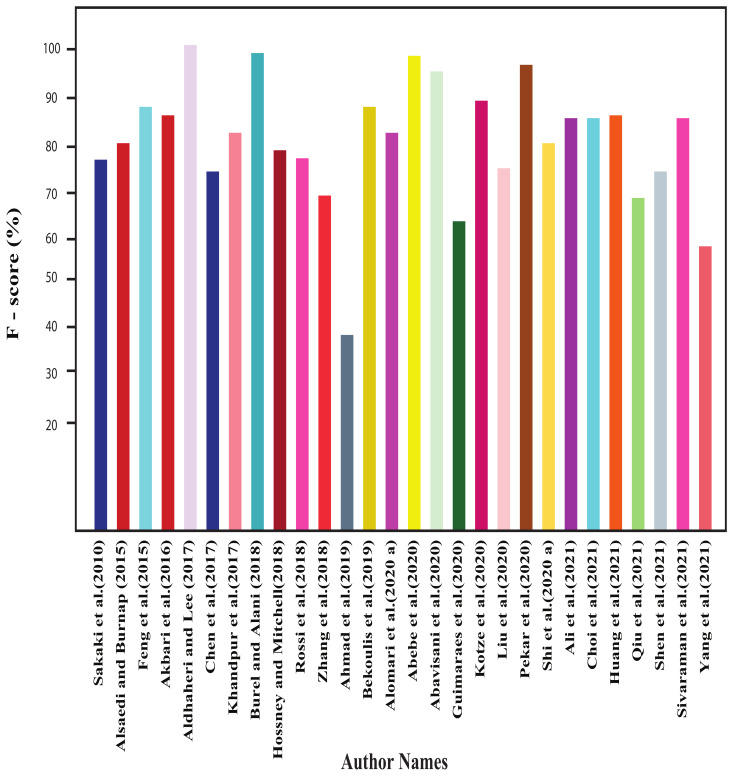
F-score-based performance comparisons of the articles. The height of each bar presents an article’s F-score, which is given in the Y-axis, and the X-axis shows the author’s name. Refs. [18,19,20,33,36,43,47,51,55,58,59,60,62,63,64,65,66,67,68,69,70,79,82,83,94,95,106,107] have used f-score for their performance analysis, and their values are presented using the bars in this figure.

**Figure 6:**
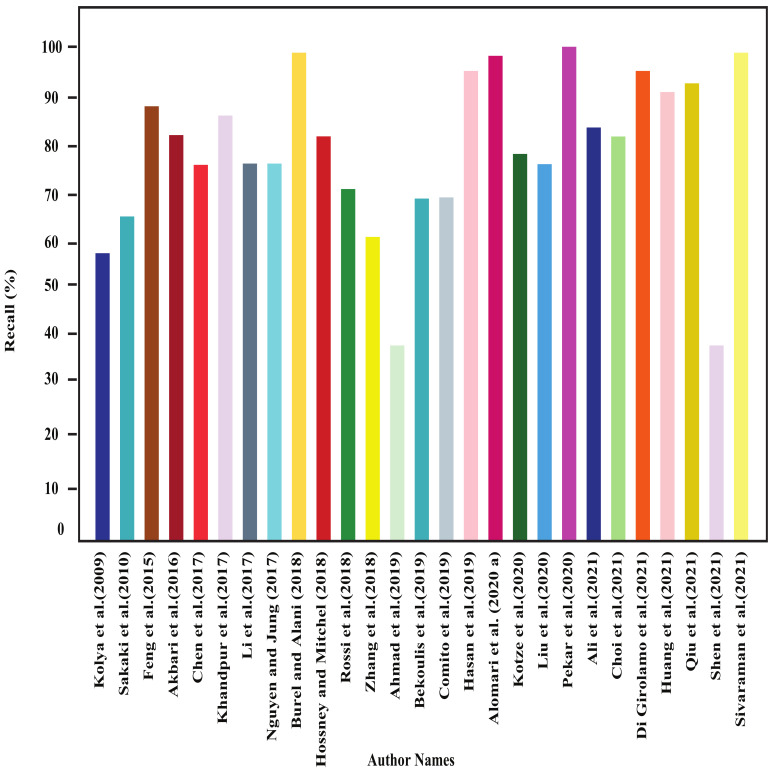
Recall-based performance comparisons of the articles. The height of each bar presents an article’s recall, which is given in the Y-axis, and the X-axis shows the author’s name. Refs. [19,20,32,33,36,45,47,53,55,59,62,63,64,65,66,67,69,70,73,75,79,83,94,97,103,106,107] have used recall for their performance analysis, and their values are presented using the bars in this figure.

**Figure 7:**
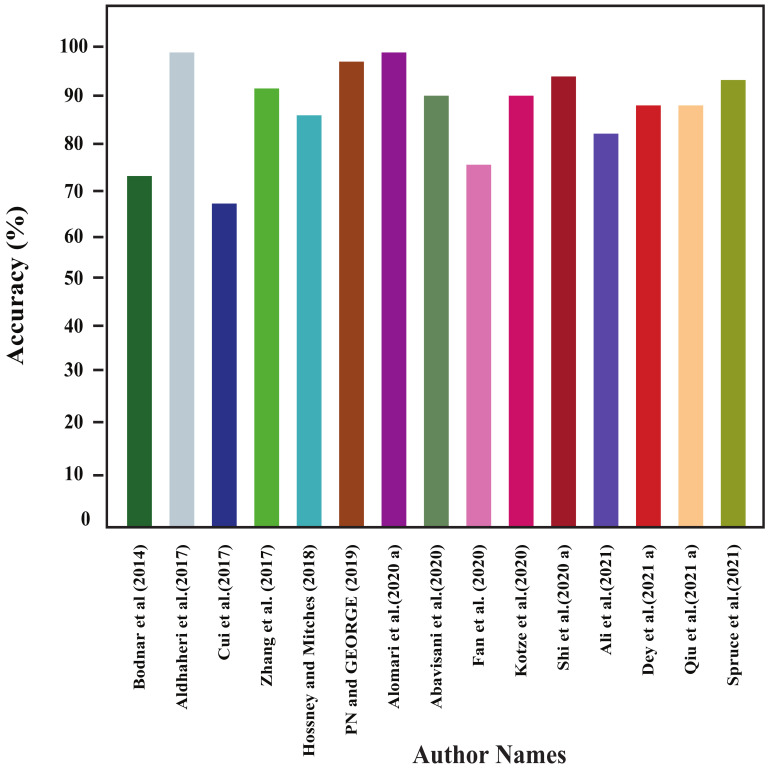
Accuracy-based performance comparisons of the articles. The height of each bar presents an article’s accuracy, which is given in the Y-axis, and the X-axis shows the author’s name. Refs. [3,4,8,19,20,32,37,48,50,58,60,61,68,69,70] have used accuracy for their performance analysis, and their values are presented using the bars in this figure.

In this section, all the review articles’ performance analysis have been performed, and their related comparisons are also shown. Figure 4, Figure 5, Figure 6 and Figure 7 show our studied articles’ precision, F-score, recall, and accuracy-based performance comparisons, respectively. In addition, Table 4, Table 5 and Table 6 describe the used datasets and associated information for the performance evaluation of shallow ML, deep ML, and other approaches, respectively.

### 4.1. Results of Shallow-Machine-Learning-Based Approaches

On detecting disruptive events, Alsaedi and Burnap [18] obtained an F measure of 80.24% using NB. They measured the precision of the eight clusters, and they are 81.39% for politics, 80.62% for finance, 79.57% for sports, 73.23% for entertainment, 76.13% for technology, 77.54% for culture, and 82.26% for disruptive events. Similarly, Dey et al. [3] found 87.5% accuracy using the BNB classification model. They also compared their model with the SVM and decision tree (DT) classifier and found an accuracy of 84.72% and 83.33%, respectively. Hossny and Mitchell [19] compared with KNN, NB, LR, and DT and gained an ROC score of up to 0.91 and an F1-score of up to 0.79. They achieved a classification accuracy of 87%, precision of 77%, and recall of 82%. Kotzé et al. [20] measured their results using both unigrams and Word2Vec models. Using word unigram (1,1), accuracy, macro-average recall, macro-average precision, macro-average F1 and micro-average F1-scores of LR were 0.899, 0.767, 0.772, 0.769, and 0.899, respectively. Using Word2vec (i.e., CBOW and skip-gram), accuracy, macro-average recall, macro-average precision, macro-average F1, and micro-average F1-scores of LR were 0.712, 0.756, 0.485, 0.514, and 0.712, respectively. Ristea et al. [21] found the AUC values for different crimes and they were assault (0.70–0.76), battery (0.74–0.79), criminal damage (0.65–0.70), motor vehicle theft (0.60–0.74), other offense (0.65–0.77), robbery (0.65–0.79), and theft (0.72–0.77).

Detecting traffic events, Suma et al. [25] applied big data and machine learning techniques for detecting spatiotemporal events. They successfully detected the Underbelly Festival, The Luna Cinema, and Notting Hill Carnival. Again, Suma et al. [27] found that for targeted event detection accuracy, the area under PR and area under ROC for LR were 78.734%, 84.706%, and 78.825%, respectively. They detected the Underbelly Festival in the South Bank. The Luna Cinema was also detected around Greenwich Park, National Trust-Morden Hall Park, and Crystal Palace Park. Notting Hill Carnival 2017 was also detected by their model. On the other hand, Alomari et al. [32] selected SVM as their main classifier. For event detection performance, they selected LR for accident and road closure events. They selected SVM for the rest of the six events’ detection. Accuracy score of SVM, LR, and NB in detecting accident, traffic condition, road closure, road damage, roadwork, social event, weather, and fire were (95%, 95%, 93%), (96%, 95%, 92%), (93%, 95%, 91%), (98%, 94%, 95%), (93%, 93%, 89%), (99%, 98%, 96%), (99%, 99 %, 96%), and (99%, 99%, 95%), respectively. Precision score of SVM, LR, and NB in detecting accident, traffic condition, road closure, road damage, roadwork, social event, weather, and fire were (92%, 94%, 96%), (97%, 95%, 95%), (97%, 95%, 97%), (98%, 91%, 99%), (89%, 88%, 91%), (99%, 99%, 95%), (99%, 98%, 95%), and (99%, 99%, 95%), respectively. Recall score of SVM, LR, and NB in detecting accident, traffic condition, road closure, road damage, roadwork, social event, weather, and fire were (96%, 95%, 89%), (96%, 95%, 90%), (88%, 95%, 84%), (98%, 98%, 92%), (97%, 98%, 87%), (99%, 98%, 98%), (99%, 99%, 96%) and (99%, 99%, 95%) respectively. F1-score of SVM, LR, and NB in detecting accident, traffic condition, road closure, road damage, roadwork, social event, weather, and fire were (95%, 95%, 93%), (96%, 95%, 92%), (93%, 95%, 91%), (98%, 94%, 95%), (93%, 93%, 89%), (99%, 98%, 96%), (99%, 99 %, 96%), and (99%, 99%, 95%), respectively.

To detect natural disaster-related events, Sakaki et al. [33] used two keywords—earthquake and shaking. For earthquakes, the average value of precision, recall, and F-score was 87.50%, 63.64%, and 73.69%, respectively. The average precision, recall, and F-score values for shaking tweets were 80.56%, 65.91%, and 72.50%, respectively. Pekar et al. [36] used three scenarios, namely, scenario 1 (i.e., training and testing on the same disaster event), scenario 2 (i.e., training and testing on the same events’ set), and scenario 3 (i.e., training on some events and testing on other events). Eventually, they considered scenario 3 for ensemble learning which was the most frequent real case, and experiments included AdaBoost, GBC, RF, DT, and disaster-based (DB-DT, DB-SVM, DB-MaxEnt). Best precision, recall, and F1-score measuring relatedness were 91.5% (DB-SVM), 100% (DB-DT, DB-MaxEnt), and 94.9% (DB-MaxEnt). The best precision, recall, and F1-score measuring informativeness were 86% (AdaBoost), 100% (DB-DT), and 86% (DB-SVM). The best precision, recall, and F1-score measuring topics were 60% (GBC), 45% (GBC), and 52% (GBC). The best precision, recall, and F1-score measuring eyewitnesses were 41% (GBC), 98% (DB-DT), and 19% (DB-SVM). Spruce et al. [37] found accuracy scores around 86–99% in different geographical areas for their task. They also found 100% accuracy in some areas, and their overall accuracy was 95% in detecting high-impact rainfall events.

In detecting foodborne disease, Cui et al. [4] obtained 32.2% accuracy for feature extraction using the TextRank algorithm maintaining the fixed context window, and 21.15% using the TF/IDF. Maintaining the dynamic context window, they obtained an accuracy of 35.3% for TextRank and 23.9% for TF/IDF. For location estimation, they experimented with 500, 1000, 1500, and 2000 tweets and obtained accuracy of 66.4%, 64.7%, 67.3%, and 64%, respectively. On the other hand, to early detect acute disease, Joshi et al. [5] observed that their system was able to detect three events before the official time (i.e., approximately 9 h before), and the cases were BreathMelbourne-StatSVM-1 in 2000, BreathMelbourne-StatSVMPerf-1 in 1000, and BreathMelbourne StatSVMPerf-1 in 2000. Similarly, their system detected events in five cases before the first news report.

For detecting journalistically relevant events, Guimarães et al. [43] mainly compared the performance of the automatic and human approaches. They found out the F1-scores of their model with different machine learning algorithms. Automatic and human approach F1-score with SVM, NB, DT, RF, GBT, and Auto MLP are (0.50, 0.28), (0.64, 0.59), (0.46, 0.28), (0.39, 0.30), (0.57, 0.55), and (0.52, 0.38), respectively. On the contrary, to track monolingual events, Kolya et al. [45] found that their total target stories were 5000, the total number of stories similar to the initial story was 56, the total number of stories similar to the initial story by the system was 39, and total number of stories correctly identified was 33. They found a recall value of 58.93% and a precision value of 84.62%.

Kumar and Sangwan [7] presented a four-steps-based rumor detection model. They categorized their dataset into two classes—rumor or non-rumor, utilizing naïve Bayes and SVM approaches.

To detect sub-events, Nolasco and Oliveira [46] conducted two experiments. The political protest database divided their results into two parts—from 16 June 2013 to 18 June 2013, a total of 14 sub-events were identified by their proposed algorithm, and from 19 June 2013 to 21 June 2013, a total of 20 sub-events were found. For the second dataset of the Zika epidemic, they divided their result into two parts—international and local. For the international part, 14 sub-events were found, and for the local part, nine sub-events were found.

Feng et al. [47] presented a real-time event detection system and obtained precision, recall, and F1-scores of 90.3%, 87.6%, and 88.9%, respectively, for all proposed features. They compared their model with the traditional 1-NN clustering and the LSH-based to establish the effectiveness of their proposed model. The precision of their proposed approach for the detected five-event clusters outperformed the other two baseline models. For real-world event detection, they obtained 17.1% precision, which also outperformed the other two models.

Zhang et al. [48] detected local events and used two datasets, named Los Angeles (LA) and New York (NY). For LA, they obtained precision, recall, and F1-score of 0.804, 0.612, and 0.695, and for NY, they obtained the scores of 0.765, 0.602, and 0.674, respectively.

Jain et al. [49] found the trustworthiness of an event and considered different event indicators, and employed a weekly supervised technique for detecting events. They gained their supervised baseline model’s precision of 95%. On the other hand, Bodnar et al. [50] assessed the veracity of an event and applied NLP and ML techniques. They used an RF classifier for the classification of their data. They found a mean ROC area of 73.14% and an accuracy of 75.41%. On the contrary, Abebe et al. [51] focused on the semantic meaning of an event and conducted four experiments. In experiment 1, wL = wS = (1 − wT)/2, and wT was varying independently between 0 and 1. Best NMI and F-score were 0.9943 and 0.9845, respectively, where wL = wS = 0.35 and wT = 0.3. In experiment 2, wT = wS = (1 − wL)/2, and wL was varying independently. Best NMI and F-score were 0.9942 and 0.9843, respectively, where wT = wS = 0.35 and wL = 0.3. In experiment 3, wT = wL = (1 − wS)/2, and wS was varying independently. Best NMI and F-score were 0.9943 and 0.9845, respectively, where wT = wL = 0.3 and wS = 0.4. In experiment 4, all weight values were varying in the range 0.25 to 0.45, and best NMI and F-score were 0.9943 and 0.9845, respectively, when wT = 0.25, wL = 0.35, and wS = 0.4. PN and GEORGE [8] proposed four-modules-based HEE models. They compared the SVM classifier and PLNN classifier in their paper, where SVM gave precision, accuracy, sensitivity, and specificity of 71.3245, 91.333, 6.45, and 99.457, and PLNN gave precision, accuracy, sensitivity, and specificity of 75.435, 96.456, 64.466, and 98.321. Gu et al. [52] extracted incidents from social media, rather than finding meaning or veracity assessment, and applied it to Pittsburgh and Philadelphia metropolitan areas. Finally, their results showed that a geocodable traffic-incident-related tweet was accountable for about 5% of all the collected tweets. Among these tweets, 60%–70% of tweets were posted by dominant users such as public agencies and media, while others were by individual users. Similarly, Nguyen and Jung [53] also extracted events and compared their model with the existing BNGram and LDA models. For their first dataset, T-REC, K-PREC, and K-REC were 0.769, 0.453, and 0.548, and for their second dataset, T-REC, K-PREC, and K-REC were 0.455, 0.652, and 0.714, respectively. Bide and Dhage [54] also detected similar events and compared the precision of their approach with existing PLSA, LDA, and EVE models. They used the average silhouette method for deciding different values of k in the k-means algorithm. Their model had a precision of 1/1, 5/5, 8/8, and 10/10 for k = 1, k = 5, k = 8, and k = 10, respectively.

### 4.2. Results of Deep-Machine-Learning-Based Approaches

Zhang et al. [55] detected traffic events and used correlation coefficient ϕ for their performance measurements of DBN. They found accuracy and precision for accident-related data and precision for non-accident related data of 0.94, 0.90, and 0.96, respectively, with ϕ = 0.05. They found accuracy and precision for accident-related data and precision for non-accident related data of 0.80, 0.65, and 0.87, respectively, with ϕ = 0.20.

For detecting meteorological events, Shi et al. [58] compared their model with CNN and single-grained capsule. Micro-average recall, micro-average precision, and micro-average F1-score of the SFMED model were 0.738, 0.862, and 0.795, respectively. In every case, the SFMED model’s values were better than CNN and single-grained capsule. SFMED outperformed the other two models in accuracy values, and SFMED, CNN, and single-grained accuracy values were 0.941, 0.897, and 0.931, respectively. On the other hand, Burel and Alani [59] detected crisis events and found precision, recall, and F1-score of CNN model (with full dataset) in predicting related or unrelated event types and information types were (0.861, 0.744, 0.797), (0.991, 0.986, 0.988), and (0.634, 0.590, 0.609), respectively. Precision, recall, and F1-score of CNN model (with sample dataset) in predicting related or unrelated event types and information types were (0.839, 0.838, 0.838), (0.983, 0.983, 0.983), and (0.610, 0.610, 0.610), respectively. Similarly, Abavisani et al. [60] detected crisis event and compared their model with compact bilinear pooling [110], compact bilinear gated pooling [111], and MMBT [112]. Accuracy, macro-F1, and weighted F1-score of SSE-Cross-BERT-DenseNet model in informativeness task, humanitarian categorization task damage, and severity task were (89.33, 88.09, 89.35), (91.94, 68.41, 91.82), and (72.65, 59.76, 70.41), respectively. These measures were calculated using Setting A (i.e., excluding the training pairs with inconsistent labels). In Setting B (i.e., informativeness task and humanitarian categorization task evaluations), the values were (90.05, 88.88, 89.90) and (93.46, 84.16, 93.35), respectively, and it outperformed other models in all aspects. In setting C, where real-world events were considered, their model’s values were (62.56, 39.82, 62.08), (84.02, 63.12, 83.55), and (86.30, 65.55, 85.93), respectively, and it outperformed other models in all aspects. For evaluation, Imran et al. [9] considered two aspects of the measure-detection rate and the hit ratio. For datasets of Joplin and Sandy, they obtained the corresponding detection rate of 78% and 41% and an HR of 90% and 78%. For both cases, Joplin showed higher accuracy. Overall, their model could identify from 40% up to 80% of disaster-relevant tweets, and finally, their approach generated an output that is 80% to 90% accurate. Fan et al. [61] offered a hybrid machine learning model for detecting disaster locations from social media data. They showed that their proposed fine-tuned BERT model obtained validation accuracy of 95.55% and test accuracy of 75.37%, which outperforms the other baseline models. Huang et al. [62] compared their model with different classifiers and showed their comparison table where text CNN obtained precision, recall, and F1-scores of 0.82, 0.92, and 0.87, respectively. For time extraction, they obtained an accuracy of 96.9%, and for location extraction, they obtained an accuracy of 94.7%.

For detecting events in the sports domain, Kannan et al. [6] showed the ROC curve of their model. For an evaluation window of 10 min, they obtained an accuracy of 80 percent in the AUROC curve, which meant that significant events were identified well within 10 min from their actual occurrence time.

Shen et al. [63] detected ADE and used two datasets—TwiMed [113] and TwitterADR [114] in their experiment. Their TwiMed dataset showed precision, recall, and F1-score of 76.14, 75.26, and 75.25, and their TwitterADR dataset showed precision, recall, and F1-score of 80.19, 71.23, and 74.49, respectively.

For multilingual event detection, Liu et al. [64] measured their detection effectiveness and found the values of precision, recall, and F1-scores of 0.6891, 0.7833, and 0.7332, respectively. For event detection, they achieved a 35.27% improvement in computing speed. In generating the event evolution graph, their model used an average of 2.5 s less time.

Ahmad et al. [65] also followed a multilingual approach and used monolingual word embedding as input features in their first experiment, where they obtained precision, recall, and F1-scores of 0.32, 0.25, and 0.25 for Hindi; 0.22, 0.18, and 0.18 for Bengali; 0.18, 0.21, and 0.18, for English respectively. Multilingual word embeddings were used as input features in their second experiment. They obtained precision, recall, and F1-score of 0.32, 0.25, and 0.26 for Hindi; 0.33, 0.25, and 0.26 for Bengali; 0.33, 0.29, and 0.28 for English, respectively. For their third experiment, multilingual word embeddings were used where they obtained precision, recall, and F1-scores of 0.40, 0.37, and 0.36 for Hindi; 0.35, 0.29, and 0.30 for Bengali; 0.43, 0.38, and 0.39 for English, respectively.

Sub-events follow events, and Bekoulis et al. [66] concentrated on this. They compared both strategies (i.e., the relaxed evaluation and the bin-level evaluation), with and without chronological LSTM in terms of precision, recall, and F1-scores, and obtained the highest F1-score of 86.59% for the Tweet-AVG model of relaxed evaluation. They concluded that their proposed binary classification baseline model showed an outstanding performance that exceeded the state-of-the-art approaches with an F1-score of 76.16% and 75.65% for macro and micro levels, respectively, in sub-event detection.

Chen et al. [67] tracked and detected online events. They measured precision, recall, F1, and DERate, and their found values were 0.800, 0.774, 0.750, and 0.063, respectively. They also compared their model with PS [115], TS [116], and MABED model [117] and in every case, their model outperformed the three other models. On the other hand, Aldhaheri and Lee [68] detected temporal events. They showed precision, recall, accuracy, and F1-scores for different regions to show the impact of image downsampling on their proposed model’s performance and obtained the highest accuracy of 99.3%. Qiu et al. [69] proposed a single-pass clustering model and showed that their approach achieved an NMI of 0.86, ARI of 0.69, and F1-score of 0.70, which ultimately showed the effectiveness of their proposed approach. Ali et al. [70] proposed sentence-level multiclass event detection and found that DNN’s accuracy, macro-average precision, macro-average recall, and macro-average F1-score were 0.84, 0.84, 0.84, and 0.84, respectively. RNN’s accuracy, macro-average precision, macro-average recall, and macro-average F1-score were 0.81, 0.82, 0.80, and 0.81, respectively. CNN’s accuracy, macro-average precision, macro-average recall, and macro-average F1-score were 0.80, 0.82, 0.75, and 0.78, respectively. They also compared their approach with machine learning algorithms. KNN’s accuracy was 0.78, DT’s accuracy was 0.73, MNB’s accuracy was 0.70, LR’s accuracy was 0.80, RF’s accuracy was 0.80, and SVM’s accuracy was 0.73.

### 4.3. Results of Rule-Based Approaches

For detecting and preventing terrorist events, Alhalabi et al. [71] proposed an anti-terrorism operation algorithm. They combined the text stream and image stream results and calculated the probability of suspicious acts that ultimately fulfilled their goal of detecting terroristic behavior.

Li et al. [73] detected real-time events. They processed a tweet in 6 microseconds. They compared their model with UMass [118,119] and LSH [120]. B-Cubed values of their model, LSH, and UMass were 0.4, 0.18, and 0.23, respectively. NMI values of their model, LSH, and UMass were 0.78, 0.52, and 0.71, respectively. For the rule-based temporal module, they found precision and recall values of 96.3% and 77.5%, respectively. Girolamo et al. [75] also detected real-time events. They detected UFC229 and Ronaldinho events. Their database contained 10,412 tweets about UFC229, and it detected the event 30 min before the starting of the event on October 6 at 8 p.m. A total of 836 tweets about Ronaldinho were in the system. It detected 2 hours after the event began. They compared their model with SFPM [76], BNGRAM, BEaTS [97], Doc-P [121], and EHG [122].

Saeed et al. [77] detected events from Twitter data. They found keyword precision for FA Cup Final, Super Tuesday, and US Election of 0.545, 0.750, and 0.423. They also found the topic-recall of the three datasets. The WDHG model identifies all the topics for the FA Cup dataset when K = 20. For the Super Tuesday dataset, WDHG outperforms other models when K > 50. The WDHG outperforms the other nine models for the US Election dataset when K > 2.

### 4.4. Results of Other Approaches

Ansah et al. [78] detected disruptive events. They found that their model detected 96% of ground truth protesting events (with FP dataset) and for that, a time window of 60 min was used. Topic intrusion, topic coherence, and precision values of SensorTree, TweetInfo, tLDA, EventTweet, and KwFreq were (0.22, 0.50, 0.70, 0.72, 0.80), (0.85, 0.50, 0.30, 0.55, 0.20), and (0.92, 0.62, 0.40, 0.60, 0.30), respectively. SensorTree outperformed the other models in all respects. On the other hand, for data breach events, the typed DQE (dynamic query expansion) method of Khandpur et al. [79] obtained precision, recall, and F1-score of 0.74, 0.68, 0.71, the baseline 1 method obtained precision, recall, and F1-score of 0.52, 0.93, 0.67, and the baseline 2 method obtained precision, recall, and F1-score of 0.21, 0.20, 0.20. For DDoS events, the typed DQE method obtained precision, recall, and F1-score of 0.80, 0.85, 0.82, the baseline 1 method obtained precision, recall, and F1-score of 0.72, 0.48, 0.58, and the baseline 2 method obtained precision, recall, and F1-score of 0.01, 0.02, 0.01. For account hijacking events, the typed DQE method obtained precision, recall, and F1-score of 0.99, 0.45, 0.61, the baseline 1 method obtained precision, recall, and F1-score of 0.99, 0.48, 0.65, and the baseline 2 method obtained precision, recall, and F1-score of 0.01, 0.01, 0.01.

In detecting traffic events, Alomari et al. [82] found out that the highest number of tweets related to events in Jeddah were posted at 22 (i.e., 10 p.m.). It decreased after three and was lowest at 8. In Makkah, the tweet rate was always high but low only during Al-Fajr prayer and Al_Dhuhr prayer time (5–12). They also found the most mentioned places in Jeddah and Makkah. The top three events detected in Jeddah were accident, fire, and inauguration. The top detected events in Makkah were rain and accidents.

Rossi et al. [83] detected disaster events. In order to test the effectiveness of their informativeness classification, they took the CrisisLexT26 database, which consisted of the tweets of 26 emergencies [123]. To evaluate the performance of their model, they used different subsets of this database and finally obtained overall effectiveness of around 70%.

To support pharmacovigilance, Rosa et al. [89] analyzed social media data. They found that 91% extracted correlations were reliable with a residual error of ±4%.

In detecting city events, Anantharam et al. [90] obtained a precision of around 94% for CRF model creation. The baseline model showed a precision of around 91%. They extracted 1042 city events, and 454 of them co-existed with the 511.org report.

For the detection of sub-events, Arachie et al. [10] generated 40 clusters for Hurricane Harvey and 50 clusters for Nepal Earthquake. They compared their method with DEPSUB and MOAC+NV methods. For Hurricane Harvey, among 795,461 unlabeled tweets and 4000 labeled tweets, their model identified 769,670 unique noun–verb pairs and 27,122 phrases. Their method had 796,792 candidate sub-events, while the DEPSUB had 769,670. For Nepal Earthquake, among 635,150 unlabeled and 3479 labeled tweets, their model identified 577,914 unique noun–verb pairs and 36,980 phrases. Their method had 614,894 candidate sub-events, while the DEPSUB had 577,914.

In order to detect real-time events, Fedoryszak et al. [93] proposed a framework. They finally generated a production application that resolved diverse product use cases and enhanced the user-examination-related metrics of continuous events.

For detecting local events, Choi et al. [94] considered OnlyTweet as their approach without relevant documents. Precision of TBEM [19], GTBEM [48], OnlyTweet, and the proposed method were 0.42, 0.38, 0.64, and 0.78, respectively. The recall values of GTBEM, TBEM, OnlyTweet, and the proposed model were 0.36, 0.40, 0.61, and 0.83. F-score of TBEM, GTBEM, OnlyTweet, and the proposed method were 0.41, 0.37, 0.62, and 0.85.

Yang et al. [95] proposed an event discovery approach. They compared their model with SDR, DGNMF, LLRR, LSDT, and SMDR. In the X->Y (using seed samples from the X domain for detecting events in the Y domain) scenario, NMI and F1-scores of ICLR were 0.7932 and 0.5748, respectively, and ICLR outperformed other baseline models in this scenario. In the Y->X scenario, NMI and F1-scores of ICLR were 0.5363 and 0.2207, respectively. SCLR outperformed other models in NMI scores, and F1-score, SMDR (F1 = 0.2432), outperformed SCLR. Comito et al. [97] proposed an online algorithm. They found event recall, keyword recall, and keyword precision on the three datasets, namely, FA Cup, Manhattan, and the US Election. For the FA Cup dataset, BEaTS event recall, keyword recall, and keyword precision were 0.61, 0.65, and 0.59, respectively. For the US election dataset, they were 0.67, 0.55, and 0.57, respectively. For the Manhattan dataset, they were 0.70, 0.72, and 0.69, respectively. For detecting events, Gao et al. [98] compared their model with candidate-ranking (CR) [124] and CLASS-SVM (CS) [125] methods. For textual content’s event detection, their proposed method showed improvements of 40.9%, 46.1%, 43.4%, and 19.4% for recall, precision, F-score, and ANMRR values, respectively (compared to the CR method). The model showed 50.7%, 55.5%, 53.0%, and 20.9% for recall, precision, F-score, and ANMRR values, respectively (compared to the CS method). For visual content to detect events, they found around 20% improvements in all the evaluation metrics. Proposing a novel model, Shi et al. [100] compared their model with independent cascade (IC), bursty event detection (BEE), EVE, and HEE. Influence score of BEE+IC, EVE+IC, HEE+IC, and EDMP in the first, second, and third propagation are (78,78, 82, 82), (92, 95, 96, 110), and (82, 81, 84, 226), respectively, where it can be seen that EDMP outperforms other models. Dong et al. [101] detected real-world events. These events included OWS protests at Zuccotti Park, Union Square, and Foley square. They also detected the Raise Cache tech event and the Mastercard free lunch promotion event. Similarly, Hasan et al. [103] proposed a practical approach. For experimental results, they used the Events2012 corpus and a collection of 506 ground-truth events. For different parameters, such as M value, ts${}_{i}$ value, Q value, t${}_{ent}$ value, t${}_{lcs}$ value, and g${}_{ev}$ value, they obtained the highest recall and precision of 0.96 and 0.80, 0.96 and 0.82, 0.96 and 0.83, 0.96 and 0.85, 0.96 and 0.89, and 0.96 and 0.89, respectively, and as a result, they determined the optimal parameter setting for their system which had a recall of 0.96 and precision of 0.89. A recall of 0.96 was found for ground truth events. Again, Valkanas and Gunopulos [104] presented an effective approach. On average, their proposed method took 3.36 ms, 0.35 ms, and 0.001 millisecond times for location extraction, classification, and event extraction, respectively. Overall, their approach took a total of 3.72 ms (average) time. Likewise, Sun et al. [105] detected events. They compared their model with PLSA and found that their method outperformed the PLSA method for every topic. In PLSA for the “The Nepal Earthquake” topic, the precision was 10/10, 11/20, and 13/30 for the top-10, top-20, and top-30 posts, respectively, whereas EVE’s precision for the same topic was 10/10, 15/20, and 21/30, respectively. Again for “The Explosion at Fujian Plant” topic, precision of PLSA for top-10, top-20, and top-30 posts were 10/10, 19/20, and 25/30, and for EVE, these values were 10/10, 20/20, and 28/30. On the other hand, Sivaraman et al. [106] initiated a unique parameter. They compared their model with Twitinfo, and their model obtained precision, recall, and F1-score of 0.727, 0.774, and 0.75. Although the precision value of their model was lower than the Twitinfo model, their recall and F1-score values outperformed the Twitinfo model. For proving the generality of their model, they also tested it with the ICWSM dataset and obtained the precision, recall, and F1-score of 0.7181818182, 0.9875, and 0.8315789474. On the contrary, Akbari et al. [107] detected wellness events. They compared their proposed model with various state-of-the-art approaches such as Alan12, SVM, Lasso, GL-MTL, and TN-MTL and obtained the best F1-score of 84.86% for gMTL. Again, Zakharchenko et al. [108] received a qualitative result which, after analyzing the data, demonstrated that their first hypothesis (H1) was true. They divided their disclosure analysis of the publications into three categories. Their result showed that the interrelation between “media quality” and the relative attention of media for the first type of publication mentioned is 0.043, which is insignificant. After their experiment, Pomytkina et al. [109] found that the percentage of students that do not feel irritated while bullying online is 44.8% (47 people), while 27.6% (29 people) students sometimes feel irritated, 18.1% (19 people) students constantly feel irritated, and 9.5% of students (10 people) often feel irritated. Additionally, the percentage of students who believe online bullying does not exist is 9.5% (10 people). They also found the roles of students during cyberbullying, which are 21% cyberbullies (initiators), 12.7% assistants, 41.9% defenders, 12.4% victims, and 12% witnesses.

## 5. Discussion

**Figure 8:**
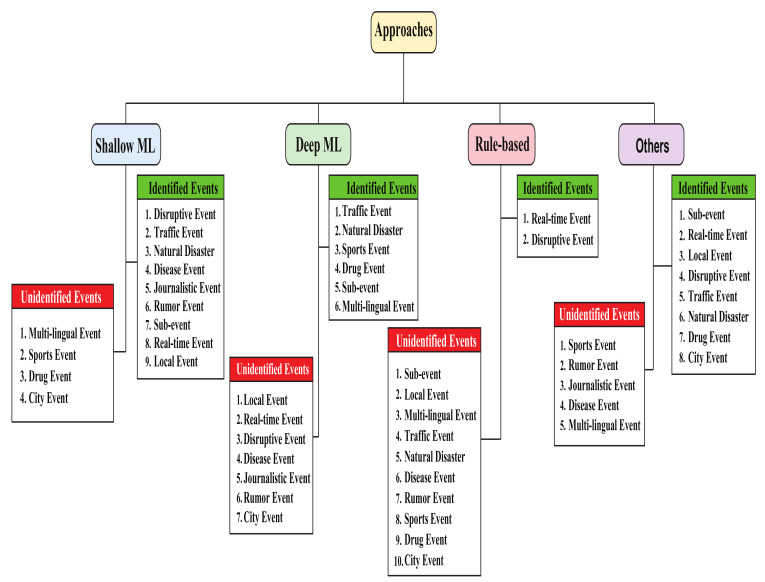
Diagrammatic presentation of obtained outcomes of our studied articles.

**Figure 9:**
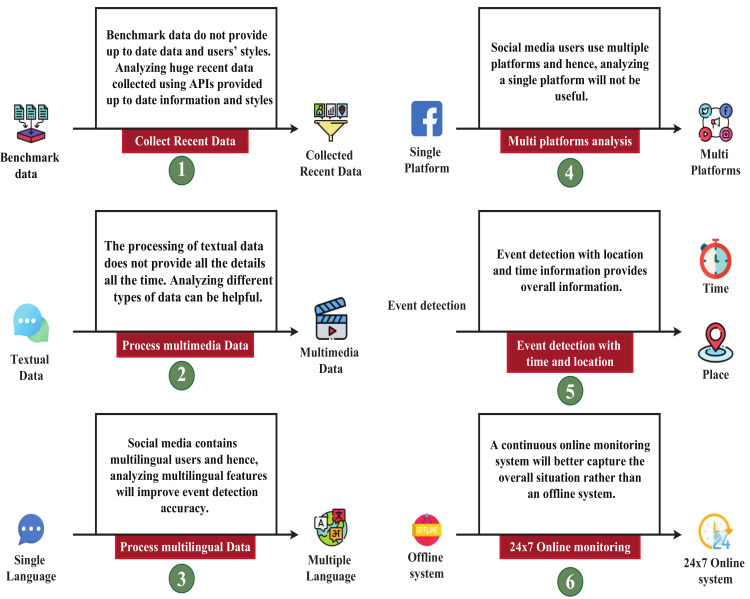
Challenges faced in event detection and solutions for their improvement.

Table 7 shows the qualitative evaluation of our studied articles using the Delphi technique. The evaluation was performed using four repetitions of questions: what was detected, was the model real-time, location, or time detected, and if the model was language-independent. The first question covers the range of events detected; the second one indicates the detection speed. The third one tells about where and when the event happened. The last question defines the range of peoples’ languages the model can analyze. A wide range of events and languages in the second and fifth columns, respectively, and “Yes” to the third and fourth column make a model robust. On the other hand, missing theses values in those columns offer scopes of improvement in the models. As evident from Table 7, not all of the studied models are language-independent; some of them are monolingual, whereas some cover two or more languages. Notably, a perfect language-independent model has not been built yet. Hence, an event detection model capable of analyzing any language is necessary. Additionally, there is a need to have more accurate event detection models with location, time, and real-time detection capacities. Moreover, a broader event detection model should identify a wide range of event types. Data from social media are being constantly utilized to identify various events. These events range from the healthcare sector to the weather. This range also includes the sports and news industries. Figure 8 shows a diagrammatic presentation of the outcomes of our studied articles. The identified and unidentified event types of our four classified approaches are shown in this diagram. The identified events are shown by green-colored boxes, which indicate the potential of a certain strategy. The red-colored boxes reflect unidentified events, indicating the approach’s limitations. According to the figure, rule-based and deep-ML-based techniques have more limitations than potential in the articles we studied. This rule-based technique may be used to identify various events, including traffic, disasters, disease outbreaks, rumor outbreaks, and so on. Local events, disruptive events, disease events, real-time events, and other events may all be detected using deep-learning-based algorithms. Shallow ML and other approaches, on the other hand, offer more potential than limitations. These potentials and additional functionalities of social media, such as nonstop monitoring systems, heterogeneous data and users, and, more importantly, global coverage of the users, will create a better event detection system. Users of any age from any part of the world can provide data, e.g., text, audio, videos, images, and so on, on these social media platforms. These facts make social media a valuable and rapid tool for event detection since social media data can be accessed instantaneously. In the challenges and future scope below, we describe how these additional functionalities can be utilized more effectively. Apart from these additional functionalities, social media also impose significant challenges, such as different types of data and their processing, efficient data collection, different writing formats and patterns of different languages, processing any languages, local dialects, real-time data processing, multiplatform data analysis, continuous monitoring of data, and accurate time and location detection. Efficient research is necessary to tackle these challenges, and further research would greatly enhance event detection tasks, which will ultimately help maintain a secure society. Figure 9 describes these lackings and their solutions. Here, we will discuss these challenges, and prospective researchers may find it promising to work in the future.

**Data Collection:** Efficient data collection is a crucial aspect of this work since peoples’ writing pattern changes over time. For this reason, up-to-date data must be collected to identify the most recent patterns in the particular languages and better detect events. Hence, using benchmark data in detecting events will not be a good idea since these data are previously collected and, in most cases, do not help in recognizing recent trends in events.

The majority of works have used benchmark data; few others have used a combination of collected and benchmark data. To better obtain the recent trends in data, recent data should be collected, and for this collection process, an efficient collection method must be employed. Some works employ a manual data collection process, which greatly hampers time. This manual collection process is very laborious since the collector must search for data on different sources, killing much time. Additionally, this process also limits the overall size of the dataset. For training underlying models, you need a large dataset, and these extensive training data ensure a better result in the testing phase since the model better captures all the scenarios. To lessen this disadvantage, efficient APIs should be employed. API ensures fast data collection and also collects enormous data in a short time.

**Heterogeneous Data and Their Processing:** The majority of the presented works are based on textual data, and this aspect has greatly limited this event detection and prediction task since social media contains a combination of textual, imagery, and video data in most cases. In some cases, the textual data might not provide event-related information, but the image or video associated with that text might provide the event-related information. Processing images and videos is also challenging since the model must recognize various noises and irrelevant information. Most importantly, the processing time must be short since we cannot spend much time obtaining real-time information. Models that incorporate these issues will surely gather more event information than the textual-based model. Hence, heterogeneous data processing models are required in this case, and future researchers might take this chance to improve this situation.

**Languages and Their Dialect-Independent Models:** Although some models work for languages such as Bengali, Hindi, Urdu, and so on, very few works consider language independence in their models. The employed model must remain language-independent to detect events in a wide range; otherwise, it will not cover other languages except for that for which it was built. A different language has its pattern of writing and vocabulary that will impact the overall detection process. For this reason, an efficient model capable of identifying multilingual features is a must. Additionally, regional dialects are prevalent nowadays in social media postings that must also be recognized to overcome this different language issue. This dialect significantly impacts the event detection process. Dialects are widespread in significant parts of the world, such as India, Bangladesh, China, etc. Hence, this dialect should be acknowledged to capture the events better. Research works have already begun to solve this issue. For example, Dey et al. [126] studied Banglish (i.e., Bengali words written in English alphabets, a regional dialect used by Bangladeshis in social media postings) in detecting events from Facebook posts. However, more efforts are needed to cover the wide range of dialects worldwide. Future researchers can improve this situation.

**Multiplatforms Systems:** Research to date has mainly focused on single-domain data for event detection. The majority of them used Facebook, Twitter, Sina Weibo, YouTube, and so on. Till now, there have been no such systems that can process multidomain data. This is because different platforms have data patterns that are difficult to process. For example, Facebook and Twitter have textual, images, and video data, whereas YouTube has video data. Researchers have processed those data separately, but there is no substantial effort to incorporate all the available social media platforms’ data. Combining multiple platforms also poses great challenges since various data types must be processed simultaneously, requiring complex and efficient models. The models will have to classify different data and apply various tools and technologies to those classified data. Additionally, there must be proper data collection tools for collecting data from different platforms since each platform has different rules and regulations. Large databases are also needed to store these data, and maintaining these databases will be another challenging issue. Researchers can work on these issues in the future to provide much better event detection systems.

**Real-Time Systems with Better Time and Location Accuracy:** Activities in social media are seen all the time, and for this reason, an instant detection system is desirable to tackle instantly generated data and detect events from those data. Additionally, real-time updates of the location and event times are also equally crucial for having accurate information about the events. However, current systems lack these factors since most of them have worked with previously collected data. Real-time data are difficult to analyze since the system has to react to the varied data styles instantaneously. An efficient real-time system with robust data analysis ability is required. Aside from the data, real-time event location and time accuracy are also poor with existing systems. Hence, this is also a prospective area for future researchers.

**Continuous Monitoring and Notifications:** Social media users are always active throughout the world, and for this reason, continuous monitoring of social media data is a must. Few works have performed this kind of study, but a well-equipped online monitoring system is required for this kind of analysis. An internet-connected system with an active API for data collection and a faster and more accurate model for data analysis is mandatory for this kind of implementation. Additionally, real-time notification systems are also essential since this system ensures awareness among the general people by providing the required information during emergencies. An email- or SMS-based notification system can be implemented in this regard.

## 6. Conclusions

This review paper focused on user-generated social media data, e.g., text, images, etc., to detect events from these contents. Due to the diversity of users and their shared contents, there has been a trend to use social media for different event-related issues such as announcing, organizing, and collecting man force or gathering for events. These events often create disruptive situations that must be detected earlier to avoid any casualties. Hence, we have tried to accumulate various event detection schemes from the last decade so that new researchers can glean ideas from them and employ new robust models to detect events. From 2009 to 2021, we systematically selected 67 research articles and presented a detailed analysis of targeted events, details on the methods used to identify those events, their performance metrics, and performance analysis. We found that most of the models follow a general approach with data collection, preprocessing, processing, classification, and visualization. To be specific:

- In the data collection phase, we noted their used datasets, collection process, and the regions from where they collected those data. Different researchers used different languages (e.g., English, Bengali, Banglish, Japanese, Hindi, Urdu, etc.) to detect events.
- In the preprocessing steps, data have been cleaned for processing. Various features have been collected and later sent for classification step in the processing phase.
- In the classification step, the articles discuss events such as disaster, traffic, disease, celebrating, protesting, religious, and many others. Their models include RF, DT, NBC, KNN, LR, LDA, SVM, LSH, BERT, LSTM, CRF, CNN, DNN, etc.
- The key performance metrics were precision, recall, accuracy, F-score, specificity, sensitivity, TP, TN, FP, FN, ROC, AUC, confusion matrix, etc.
- We found that different models perform well for different event detection. For example, for detecting a protest, NBC model performs best; for detecting disaster, bi-LSTM and CNN models perform best; for detecting disease and earthquake, SVM model works well; for detecting sports, LSH model performs better; for detecting violent events, LR model performs best.

A significant research gap remains for this huge research domain despite considerable research. There is no language- and dialect-independent system available for analyzing data. Efficient real-time systems are missing, along with facilities such as 24 × 7 monitoring and quick notification services. These are essential aspects in terms of processing social media data. Moreover, a multiplatform data analysis system is also necessary for covering a large number of online participants. A robust data collection system is required too, for collecting data from these platforms that are still missing in the current research trends.

## Figures and Tables

**Table 1:** A brief description of the selected features, studied languages, and evaluation matrices used in articles that applied shallow ML techniques.

Ref.	Selected Features	Used Language	Evaluation Matrices
[3]	Events specific words and phrases frequency, sentiment score	Bengali	Accuracy, receiver operating characteristics (ROC), area under curve (AUC)
[4]	Total followers, total users, personal description’s length, total tweets, average retweeting, average recommendations, average comments, average length of 165 tweets, time of tweet and average links in tweet	×	Accuracy
[5]	Word embeddings(in the statistical classification of health mention)	×	×
[7]	Bag of words, part-of-speech, emoticon, named entity recognition, sentiment, event, time, origin, hashtags, links and urls	×	×
[18]	Retweet ratio, hashtags, temporal, spatial and textual features(near duplicate ratio, mention ratio, retweet ration, hashtag ratio, url ratio, sentiment)	×	F measure
[19]	Word-pair frequency	English	Accuracy, ROC, recall, F
[20]	Unigram, Word2Vec	English, Africaans	Accuracy, macro-recall, macro-precision, macro-F1, micro-F1
[32]	TF-IDF	Arabic	Precision, F1, recall, and accuracy
[33]	Keywords, number of words, context	English, Japanese	Precision, recall & F-score
[43]	Text statistics (number of words, post length, word embeddings), domain (several lexicons from Oxford Topic Dictionary), sentiment (positive, negative and neutral), and entities (person, locations, organizations, and dates) were considered features. Additionally, they also counted user agreement rate deviation, user’s correct words percentage, and user’s consistency regarding news relevance for human approach. For automatic approach, identifications of people, location, date, organizations, and keywords.	×	F1
[45]	Context words, suffix and prefix of word, named entity information, first word (binary value), length of word (binary value), infrequent word (binary value), digit features, position of the word, POS information, Gazetteer list, common words—these are the features for named entity recognition	×	Recall, precision
[47]	Heuristis features such as PosTag, PosTagBefore, PosTagAfter, LabelAfter, and IsSymWord	×	Precision, recall, F1 value
[50]	Numerical features (no. of friends, tweets, favorites, followers, and favored), n-garms from the text data	×	ROC, accuracy
[51]	Temporal, spatial, and semantic	×	Normalized mutual information (NMI) and F-score measures
[52]	“*Road-name*”, “exit”, “accident”, “traffic”, “roadwork”, “lane”, “PA”, “mile”, “cleared”, and “post”	×	Confusion matrix
[53]	Occurrence score, diffusion degree, diffusion sensitivity	×	Precision, recall

**Table 2:** A brief description of the selected features, studied languages, and evaluation matrices used in articles that applied deep ML techniques.

Ref.	Selected Features	Used Languages	Evaluation Metrics
[6]	Unigrams	×	ROC curve
[9]	Word unigrams, bigrams, part-of-speech (POS) tags, binary features, and scalar features	×	Detection rate, hit ratio
[55]	Individual and paired tokens	English	Accuracy, precision
[65]	Multilingual word embedding, monolingual word embedding	Hindi, Bengali, and English	Precision, recall, F1, confusion matrix
[70]	Unigram and bigram tokens	Urdu	Accuracy, precision, recall, and F1

**Table 4:** Shallow-ML-based articles’ used datasets and their related information: presenting total datasets created, size of datasets, duration of data collection, geographical area, and their collection method.

Ref.	Size	Duration of Data Collection	Area	Collection Method
[4] 1 dataset created	80 million	August–October 2014	Beijing	Weibo API
[5] 3 datasets used	BreathMelbourne (2,672,578 tweets), CoughMelbourne (985,180 tweets), and OtherMelbourne (152,113 tweets)	2014–2016	Australia (Melbourne)	Twitter REST API
[19] 1 dataset created	6 billion words pairs	Data collected for 540 days (4 million tweets per day)	×	
[20] 1 dataset created	15 WhatsApp groups between. In total, 23,360 WhatsApp messages were retrieved in either English or Afrikaans	30 May 2018 and 18 February 2019	South Africa	×
[21] 1 dataset created	Crime incident data were downloaded from Chicago Police Department’s (Citizen Law Enforcement Analysis and Reporting) database (111,936 incidents). Twitter data (9,436,276 GPS-tagged tweets) also collected.	31 October 2012 and 14 April 2014. Twitter—31 October 2012 and 14 April 2014	Chicago	Twitter API
[27] 1 dataset created	3 million tweets	Collected from 21 August–13 September 2017	London	×
[32] 1 dataset created	2.5 million tweets (2,511,000 tweets)	23 September–7 October 2018	Saudi Arabia (Riyadh, Makkah)	Twitter REST API
[33] 3 datasets created	597 tweets for training, 621 for location & 2037 for trajectory detection		Japan	
[37] 1 dataset created	Met Office data, 44.7 million tweets	Met—1 January 2017–30 June 2017, Twitter—1 January 2017–30 June 2017	Across the world (USA, UK, Australia, Malaysia, Saudi Arabia, Angola, India, Haiti)	Met—manual Twitter—Twitter streaming API
[43] 2 datasets created	4994 Facebook posts and comments, 4994 tweets (human approach). For the automatic approach, 7853 posts from Facebook and 3831 tweets were collected	7–14 September 2016	USA	×
[45] 1 dataset created	108,305 news documents with 2,822,737 total sentences.		India	×
[46] 2 datasets used	×	Political protest of Brazil (2013), Zika epidemic dataset (2015–2016)	Brazil	built in API
[50] 4 datasets used	Boston Marathon 2012, The Dark Knight Rises 2012, bombing of the White House in 2013, bomb threat at Harvard 2013	×	USA	×

**Table 5:** Deep-ML-based articles’ used datasets and their related information: presenting total datasets created, size of datasets, duration of data collection, geographical area, and their collection method.

Ref.	Size	Duration of Data Collection	Area	Collection Method
[6] 1 dataset	Tweets of 44 games with a file size of over 6 GB	×	×	Streaming API of Twitter
[9] 2 datasets used	Joplin (206,764 tweets) and Sandy (140,000 tweets)	×	Missouri (USA), northeastern US	Twitter’s API
[55] 1 dataset created	Over 3 million tweets collected during. 584,000 geotagged tweets from Northern Virginia, 2,420,000 geotagged tweets from New York City	(January–December 2014)	Northern Virginia, New York	Twitter streaming API with geolocation filter
[58] 1 dataset created	1,123,000 Weibo posts	×	China	Sina Weibo API
[59] 1 dataset created	CrisisLexT26 dataset (28,000 tweets with 26 different crises)	2013 and 2012	×	×
[60] 1 dataset used	CrisisMMD dataset	2017	×	
[61] 1 dataset used	About 21 million tweets	Hurricane Harvey of 2017	Houston	Twitter PowerTrack API and filtering rules
[65] 1 dataset used	Total dataset 2191 documents (Hindi: 922, Bengali: 999, and English: 270	×	×	Crawling
[66] 1 dataset used	2M preprocessed tweets filtered from 6.1M collected ones	Tweets from 2010 (soccer matches) and 2014 (FIFA World Cup)	×	×
[67] 1 dataset created	9,563,979 tweets for evaluation, 33,808 event-related and 33,808 non-event-related tweets for training, for similarity metric learning, 1,000,000 tweet pairs as positive, and another 1000,000 tweet pairs as negative	Evaluation tweets were collected between 10 November 2016 and 10 December 2016.	×	Twitter public API
[68] 1 dataset	17 GB	Time period of three weeks from 17 September 2014 to 20 November 2014	×	Twitter Streaming API
[70] 1 dataset created	0.15 million (103,965) lab- eled sentences	×	×	PHP-based web crawler was used

**Table 6:** Other-approach-based articles’ used datasets and their related information: presenting total datasets created, size of datasets, duration of data collection, geographical area, and their collection method.

Ref.	Size	Duration of Data Collection	Area	Collection Method
[10] 2 datasets used	Hurricane Harvey of 2017 (795,461 distinct unlabeled tweets), Nepal Earthquake of 2015 (635,150 distinct unlabeled tweets)	×	×	×
[78] 4 datasets used	1. Free Port (FP)—200,800 tweets2. US–Ghana Military Base (USGH)—100,000 tweets3. Live exports—3,200,000 tweets4. Newcastle Harbour Blockade (NHB)—4,900,305 tweets	1. January–April 20172. 19–30 March 20183. 3–30 June 20184. February–May 2016	1. Indonesia2. Ghana3. Australia4. Australia	DS 2,3,4 collected by Twitter public API. DS 1 was provided by Data to Decisions CRC (D2DCRC)
[79] 1 dataset used	5,146,666,178 tweets	27 months: from August 2014 to October 2016	×	×
[82] 1 dataset created	×	17 May–14 June 2018	Jeddah, Makkah	REST search API for Twitter searching
[83] 1 dataset used	260k tweets	×	Italy	×
[89] 1 dataset created	20,000 tweets during 2009–2019. Four thousand papers from 1974–2019	Tweets (2009–2019), papers (1974–2019)	×	Selenium WebDriver for Twitter data collection, used official API through Europe PMC services for PubMed data collection
[90] 1 dataset created	8 million	4 months	San Francisco Bay	×
[94] 1 dataset created	Total of 119,243 tweets and 137,942 relevant documents were considered	Data collected for the whole month of August 2020	×	Twitter’s standard API
[97] FA cup, US election (2012), and 1 created dataset	×	December 2014	Manhattan (created one)	×
[98] 1 dataset used	Brand-Social-Net dataset (3 million tweets with 1.3 million images)	June–July 2012	×	×
[100] 1 dataset created	1,500,000 posts and 36,845 users	×	×	Twitter API
[105] 1 dataset used	Data consisted of 49,918 posts and 4916 users	14 April 2015 to 15 April 2015	Nepal, China	By SinaAPI
[106] 2 datasets used	3.3 M dataset (3.3 million tweets), ICWSM Dataset (1,280,000 tweets)	3.3 M dataset (from 15 January 2018 to 4 March 2018), ICWSM Dataset (from 14 December 2011 to 11 January 2012)	×	Using Twitter API

**Table 7:** Qualitative performance evaluation of our studied articles using Delphi technique.

	Round 1 Question	Round 2 Question	Round 3 Question	Round 4 Question	
**Ref**	**Detected What?**	**Was the Model Real-Time?**	**Location or Time Detected?**	**Is the Model Language-Independent?**	**Comments**
[3]	Protest, religious, celebrating	No	No	No	Real-time, location, and time detection with multilingual features would be better
[6]	Sports event	Yes	No	No	
[7]	Rumor	No	No	No	Incorporating time and location detection would be commendable
[9]	Disaster-related event	No	No	No	Introducing a multilingual model would show better performance
	Including time and location detection would make the model more efficient				
[18]	Disruptive	Yes	No	No	Location and time detection with multilingual feature would be better
[19]	Protest	Yes	No	Used multiple languages	Incorporating multiple languages is praiseworthy; should be extended with more languages
[25]	Congestion	Yes	Location only	No	Time detection with multilingual feature would be better
[33]	Earthquake	Yes	Yes	No	Multilingual detection would be better in covering worldwide events
[45]	Monolingual event tracking from newspaper corpus	No	No	No	A robust model with real-time, place, and location with various languages would be better
[52]	Traffic detection	Yes	Yes	No	Introducing a multilingual model would be great
[61]	Disaster event	No	Location only	No	Detecting time of the disaster would be excellent
[64]	General events	Yes	No	Yes	Location and time detection would result in a better model
[65]	Disaster event	No	No	multilingual	Extracting time and location of disaster would be better
[70]	Multiclass event classification	No	No	No	Considering multiple languages with real-time feature would be better
[71]	Terroristic behavior	No	Location only	Multilingual	A real-time-based model would show much better performance
[73]	Novel events	Yes	Yes	No	Multilingual approach would make the model more robust
[77]	Trending events	No	No	No	Real-time event detection would be better
[79]	Cyber-attack	No	No	No	Making the model a real-time one would be great
